# The Environmental Sensor AHR Protects from Inflammatory Damage by Maintaining Intestinal Stem Cell Homeostasis and Barrier Integrity

**DOI:** 10.1016/j.immuni.2019.05.024

**Published:** 2019-06-18

**Authors:** Amina Metidji, Sara Omenetti, Stefania Crotta, Ying Li, Emma Nye, Ellie Ross, Vivian Li, Muralidhara R. Maradana, Chris Schiering, Brigitta Stockinger

(Immunity *49*, 353-362.e1–e5; August 21, 2018)

## Main Text

In the originally published Figure 6, the legend describing panel B was incorrect. The error occurred because the text from the legend describing Figure 5B had accidentally been copied. The corrected legend now appears online and in print here.

Figure 6. Dietary AHR Ligands Can Halt Progression of Tumorigenesis

(A) Villin-cre^*R26Cyp1a1*^ were injected with 10 mg/kg of AOM followed with one cycle of 1% DSS on standard chow diet. For the second cycle of DSS, mice were put either on a purified diet or I3C diet until the end of the experiment.

(B) Number of colon tumors in Villin-cre^*R26Cyp1a1*^ n = 10 mice, fed purified or I3C diet after AOM/DSS treatment.

(C) Representative image of colon after AOM/DSS treatment.

(D and E) Size (D) and score (E) of tumors in Villin-cre^*R26Cyp1a1*^ mice fed purified or I3C diet.

(F) Representative images of hematoxylin and eosin (H&E) colon tumors in VillinCre^R26Cyp1a1^ mice fed purified or I3C diet. Error bars, mean + SEM. ^∗^p < 0.05, ^∗^^∗^p < 0.01, ^∗^^∗^^∗^p < 0.001, as calculated by paired t test.

Furthermore, we were made aware of an error in Figure 2A in which a partial overlap of the panel for *Ahr*^*−/−*^ is repeated in the image for R26^Cyp1a1^. This mistake happened during selection of the example panels from a multitude of images. Although it has no bearing on the validity of the data in this figure because panel A was purely illustrative to show different growth characteristics in AHR mutant colon organoids, we have now replaced this panel with a new panel for R26^Cyp1a1^ in Figure 2A. The corrected figure now appears online and in print here. The authors sincerely apologize for any confusion that these errors might have made for the readers.

**Figure 2:**
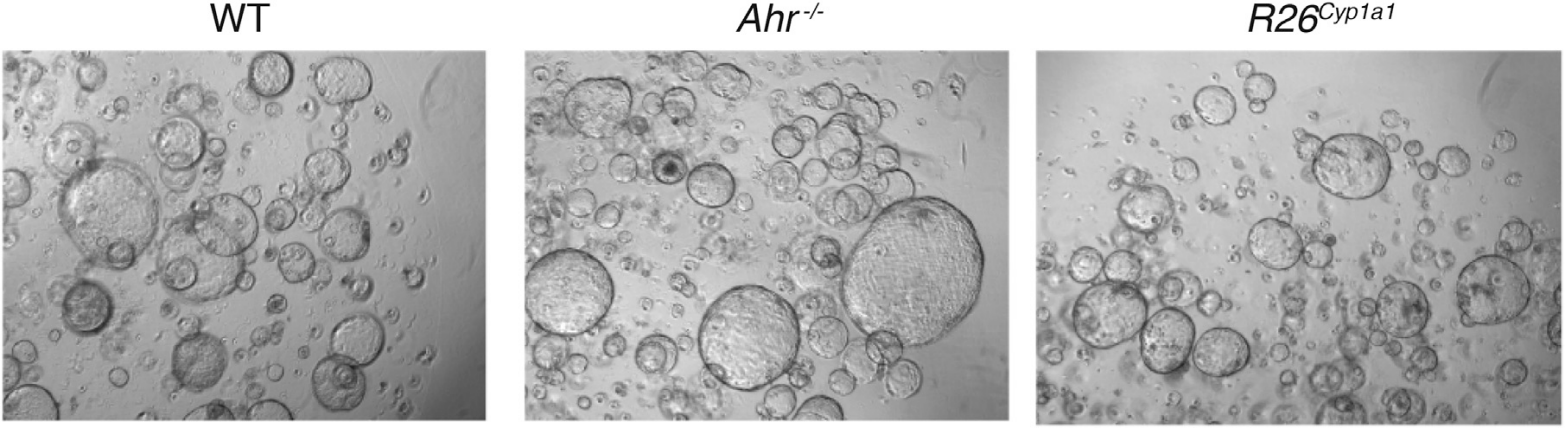
AHR Pathway Is Required for Epithelial Cell Differentiation from Crypt Stem Cells (corrected)

**Figure 2:**
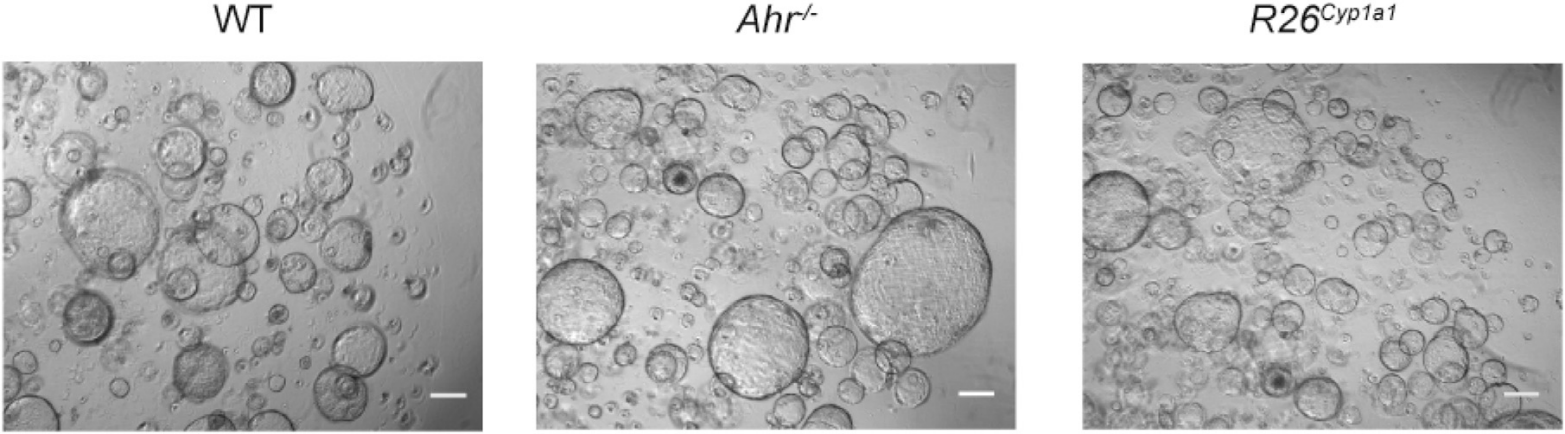
AHR Pathway Is Required for Epithelial Cell Differentiation from Crypt Stem Cells (original)

