# Supplementary material for: The Environmental Sensor AHR Protects from Inflammatory Damage by Maintaining Intestinal Stem Cell Homeostasis and Barrier Integrity
Source: Immunity. 2018 Aug 21;49(2):353–362.e5. doi: 10.1016/j.immuni.2018.07.010 (PMC6104739; doi:10.1016/j.immuni.2018.07.010)
Supplement: Document S1. Figures S1–S3 [file mmc1.pdf]

Immunity, Volume *49*

Supplemental Information

The Environmental Sensor AHR Protects from Inflammatory Damage by Maintaining Intestinal Stem Cell Homeostasis and Barrier Integrity

Amina Metidji, Sara Omenetti, Stefania Crotta, Ying Li, Emma Nye, Ellie Ross, Vivian Li, Muralidhara R. Maradana, Chris Schiering, and Brigitta Stockinger

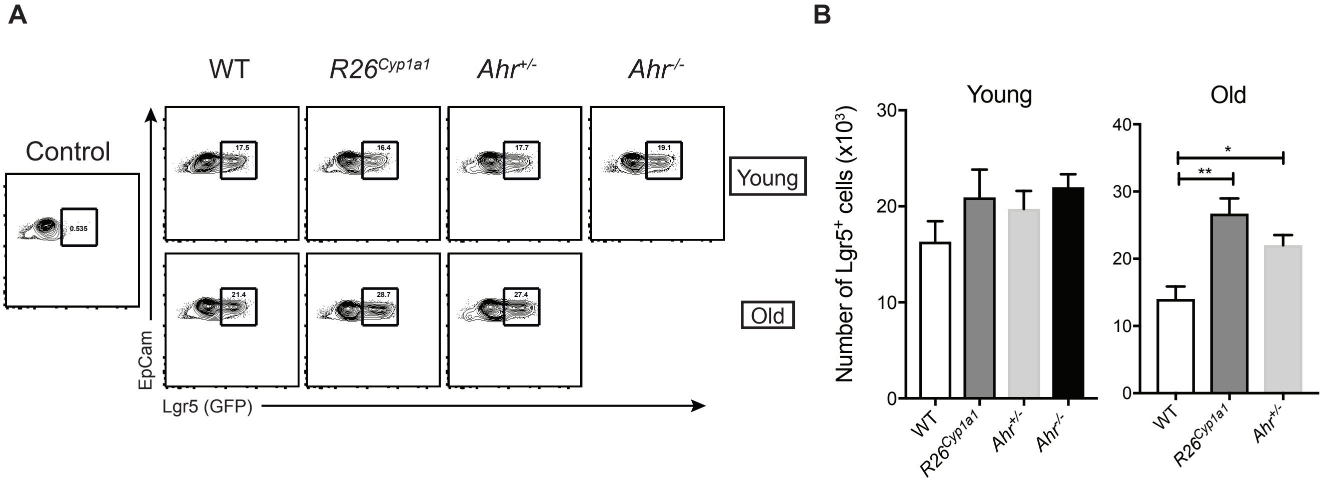

Figure S1. Expansion of Lgr5$^+$ cells with age, related to Figure 3.
(A) Flow cytometry analysis of Lgr5 expression in EpCam$^+$ cells from the colon of the indicated mice at steady state.
(B) Percentage of Lgr5$^+$ cells at steady state in young (5-8 weeks of age) and old (14-16 weeks of age) mice.
Error bars, mean + s.e.m. * $P < 0.05$, ** $P < 0.01$, ***$P < 0.001$, as calculated by one-way ANOVA with Tukey post-test.

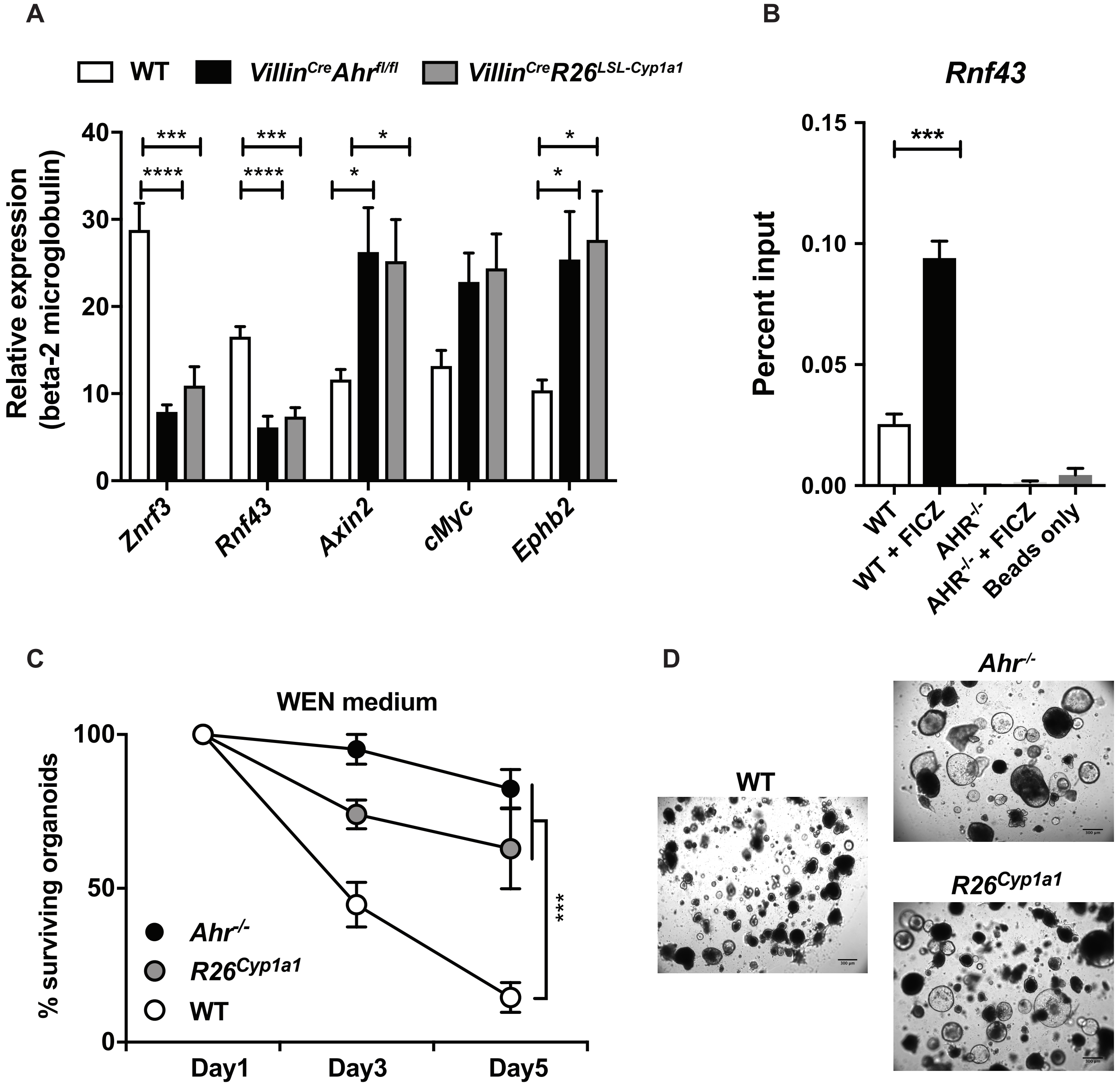

Figure S2. Dysregulated feedback signaling in the Wnt-β-Catenin pathway of AHR mutant mice, related to Figure 4.
(A) qPCR analysis of WNT negative regulators (*Znrf3* and *Rnf43*) and WNT target genes (*Axin2*, *cMyc*, and *Ephb2*) in the colon of 14 weeks old mice (WT, n=5; $Villin^{Cre}Ahr^{fl/fl}$, n=5; $Villin^{Cre}R26^{LSL\text{-}Cyp1a1}$, n=5) at steady state. (B) Chromatin immunoprecipitation (ChIP) analysis of AHR interaction with *Rnf43* promoter in WT, WT treated with FICZ, $Ahr^{-/-}$, and $Ahr^{-/-}$ treated with FICZ in stem cells organoids. (C) Percentage surviving organoids following culture in WEN medium (without R-spondin1) over 5 days calculated using OrganoSeg software. (D) Representative images of WT, $Ahr^{-/-}$ and $R26^{Cyp1a1}$ organoids after 5 days in WEN medium. Error bars, mean + s.e.m. *P < 0.05, **P < 0.01, ***P < 0.001, as calculated by one-way ANOVA with Tukey post-test.

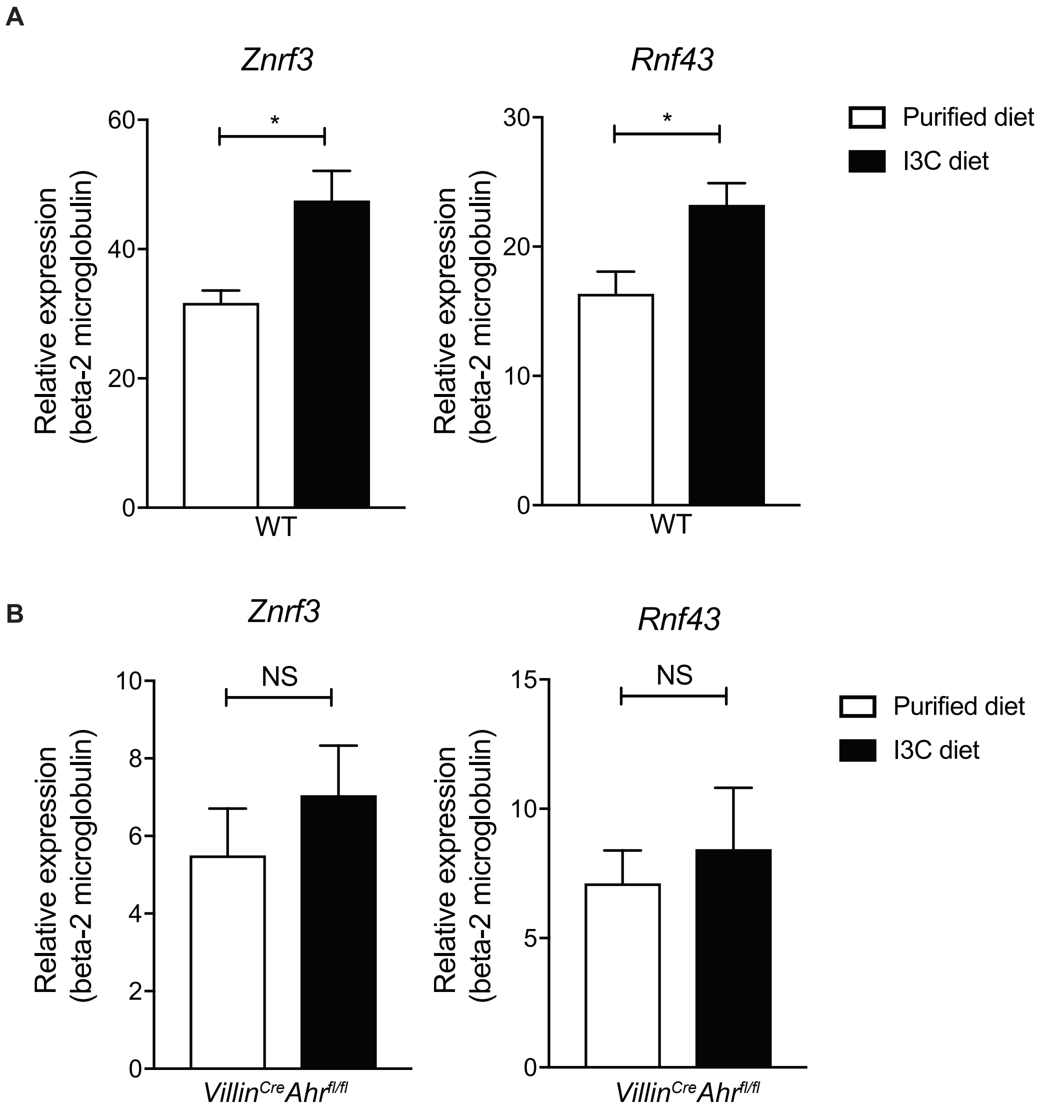

Figure S3. I3C diet increases WNT pathway regulator expression in WT but not *Villin*^{Cre}*Ahr*$^{fl/fl}$ mice, related to Figure 5. (A) qPCR analysis of WNT negative regulators (*Znrf3* and *Rnf43*) in the colon of WT, n=5 after AOM/DSS fed purified or I3C diet. (B) qPCR analysis of WNT negative regulators (*Znrf3* and *Rnf43*) in the colon of *Villin*^{Cre}*Ahr*$^{fl/fl}$, n=5 after AOM/DSS fed control or I3C diet. Error bars, mean + s.e.m. * $P < 0.05$, ** $P < 0.01$, *** $P < 0.001$, as calculated by unpaired t-test.
