# Supplementary material for: The Environmental Sensor AHR Protects from Inflammatory Damage by Maintaining Intestinal Stem Cell Homeostasis and Barrier Integrity
Source: Immunity. 2018 Aug 21;49(2):353–362.e5. doi: 10.1016/j.immuni.2018.07.010 (PMC6104739; doi:10.1016/j.immuni.2018.07.010)
Supplement: Document S2. Article plus Supplemental Information [file mmc2.pdf]

Immunity

Article

The Environmental Sensor AHR Protects from Inflammatory Damage by Maintaining Intestinal Stem Cell Homeostasis and Barrier Integrity

Graphical Abstract

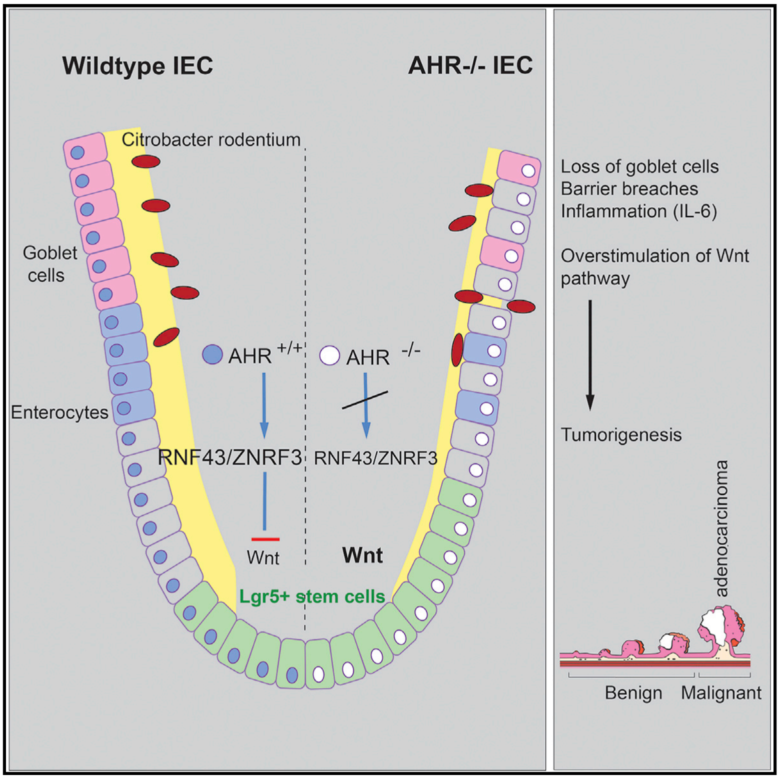

Highlights

- AHR deficiency in IECs promotes inflammation and colon tumorigenesis
- AHR activation regulates intestinal crypt stem cell differentiation
- AHR controls negative regulators of the Wnt-β-catenin pathway
- A diet rich in AHR ligands can prevent or halt tumorigenesis

Authors

Amina Metidji, Sara Omenetti, Stefania Crotta, ..., Muralidhara R. Maradana, Chris Schiering, Brigitta Stockinger

Correspondence

c.schiering@lms.mrc.ac.uk (C.S.), brigitta.stockinger@crick.ac.uk (B.S.)

In Brief

Metidji et al. show that deletion of *Ahr* in intestinal epithelial cells results in a defective barrier and unrestricted proliferation of intestinal stem cells (ISCs), culminating in malignant transformation. Activation of AHR by dietary ligands guards the ISC niche and maintains intestinal barrier homeostasis.

Metidji et al., 2018, Immunity *49*, 353–362
August 21, 2018 © 2018 The Author(s). Published by Elsevier Inc.
https://doi.org/10.1016/j.immuni.2018.07.010

CellPress

The Environmental Sensor AHR Protects from Inflammatory Damage by Maintaining Intestinal Stem Cell Homeostasis and Barrier Integrity

Amina Metidji,[1] Sara Omenetti,[1] Stefania Crotta,[1] Ying Li,[1] Emma Nye,[1] Ellie Ross,[2] Vivian Li,[1] Muralidhara R. Maradana,[1] Chris Schiering,[1,3,*] and Brigitta Stockinger[1,*]
[1]The Francis Crick Institute, London NW1 1AT, UK
[2]The Royal Veterinary College, University of London, London, UK
[3]Present address: Medical Research Council London Institute of Medical Sciences, Hammersmith Hospital, Imperial College, London W12 0NN, U.K
*Correspondence: c.schiering@lms.mrc.ac.uk (C.S.), brigitta.stockinger@crick.ac.uk (B.S.)
https://doi.org/10.1016/j.immuni.2018.07.010

SUMMARY

The epithelium and immune compartment in the intestine are constantly exposed to a fluctuating external environment. Defective communication between these compartments at this barrier surface underlies susceptibility to infections and chronic inflammation. Environmental factors play a significant, but mechanistically poorly understood, role in intestinal homeostasis. We found that regeneration of intestinal epithelial cells (IECs) upon injury through infection or chemical insults was profoundly influenced by the environmental sensor aryl hydrocarbon receptor (AHR). IEC-specific deletion of *Ahr* resulted in failure to control *C. rodentium* infection due to unrestricted intestinal stem cell (ISC) proliferation and impaired differentiation, culminating in malignant transformation. AHR activation by dietary ligands restored barrier homeostasis, protected the stem cell niche, and prevented tumorigenesis via transcriptional regulation of of *Rnf43* and *Znrf3*, E3 ubiquitin ligases that inhibit Wnt-β-catenin signaling and restrict ISC proliferation. Thus, activation of the AHR pathway in IECs guards the stem cell niche to maintain intestinal barrier integrity.

INTRODUCTION

The intestinal epithelium constitutes a single-layer barrier that separates the mucosal immune system from trillions of commensal bacteria. Interactions between intestinal epithelial cells (IECs), immune cells, and the microbiota underlie the maintenance of intestinal homeostasis in steady state as well as upon perturbation by infection. The integrity of the intestinal barrier has substantial implications for health even beyond the intestine. Numerous genetic loci are known to contribute to the development of inflammatory bowel diseases such as Crohn's disease or ulcerative colitis and the genetic susceptibility for disease is well documented (Liu et al., 2015). However, environmental factors including smoking, diet, and use of antibiotics play a significant role in the etiology of intestinal disorders, and the molecular mechanisms underlying their impact remain poorly defined.

The environmental sensor AHR is highly expressed at barrier sites such as the skin, lung, and gut. Although the AHR was originally described as a receptor for dioxin and other xenobiotics, it is now clear that physiological AHR ligands such as dietary components and tryptophan metabolites (Denison and Nagy, 2003; Hubbard et al., 2015; Lamas et al., 2016; McMillan and Bradfield, 2007; Moura-Alves et al., 2014; Zelante et al., 2013) serve to drive beneficial functions of AHR in the immune system as well as in non-hematopoietic cells. In the context of intestinal homeostasis, *Ahr* deficiency has detrimental consequences associated with loss of intraepithelial lymphocytes and ILC3 and absence of IL-22 production (Kiss et al., 2011; Lee et al., 2011; Li et al., 2011; Qiu et al., 2012). An important aspect of AHR activation is the necessity for negative feedback regulation as prolonged stimulation has detrimental effects (Andersson et al., 2002; Mitchell and Elferink, 2009). AHR activation induces expression of a family of cytochrome P450 enzymes (CYP1 family), which metabolize AHR ligands, thereby terminating the stimulus (Schmidt and Bradfield, 1996). In support of this, we recently showed that selective overexpression of CYP1A1 in IECs ($Villin^{Cre}R26^{LSL\text{-}Cyp1a1}$ mice) acts as a metabolic roadblock leading to insufficient AHR ligand supply to mucosal immune cells, thereby compromising ILC3- and Th17 cell-mediated immunity to enteric infection (Schiering et al., 2017). However, the expression of CYP1A1 along the crypt-villus axis in response to dietary AHR ligand exposure strongly suggests a role for AHR in IEC function beyond regulation of ligand supply to the host.

The rapid regeneration of the intestinal epithelium is a highly coordinated process that is fueled by the proliferation of LGR5-expressing intestinal stem cells (ISCs) located at the bottom of each crypt (Barker et al., 2007). The Wnt-β-catenin pathway is crucial for the proliferation and maintenance of ISCs and is tightly regulated by E3 ubiquitin ligases RNF43 and ZNRF3, which target WNT receptors for degradation (Koo et al., 2012). Aberrant Wnt-β-catenin activation is a hallmark of colorectal cancers, highlighting the importance of this pathway in intestinal homeostasis (Novellasdemunt et al., 2015).

Utilizing mouse models as well as intestinal organoid cultures, we found that AHR acts directly on IECs to restrict excessive proliferation of ISCs through regulation of *Rnf43* and *Znrf3* expression. As a consequence, *Ahr* deficiency in IECs compromised

This is an open access article under the CC BY license (http://creativecommons.org/licenses/by/4.0/).

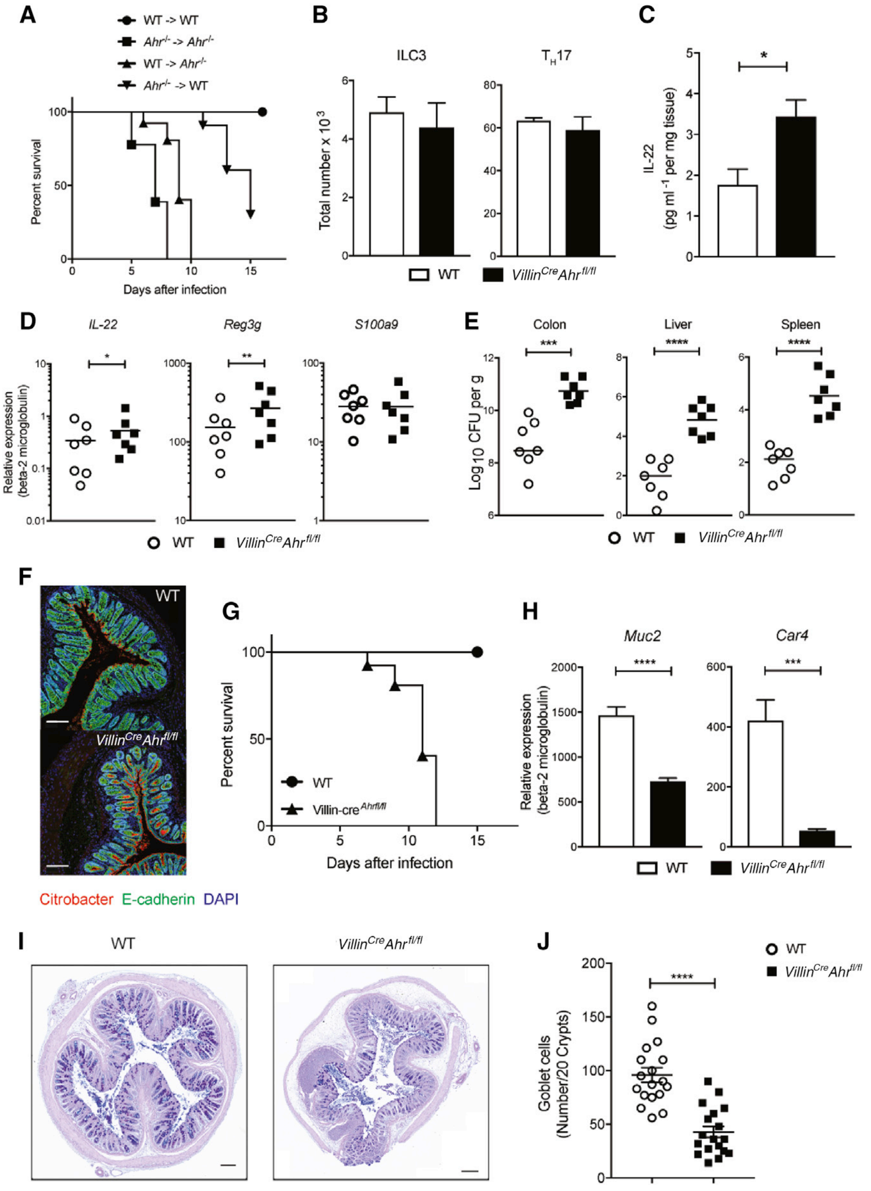

Figure 1. *Ahr* Deficiency in IEC Impairs Resistance to *Citrobacter rodentium*

(A) Survival plot of bone marrow chimeras infected with *C. rodentium* (WT→ WT; $Ahr^{-/-}$ → WT; $Ahr^{-/-}$ → $Ahr^{-/-}$; WT → $Ahr^{-/-}$); n = 5 per group.

(B) Absolute numbers of colonic RORγt^{+} ILC3 and IL-17A-producing TCRβ^{+}CD4^{+} T cells (WT, n = 7; $Villin^{Cre}Ahr^{fl/fl}$, n = 7) at day 7.

(C) IL-22 protein content in colon explant cultures (WT, n = 12; $Villin^{Cre}Ahr^{fl/fl}$, n = 12). Data represent pooled results of at least two independent experiments.

(D) qPCR analysis of antimicrobial (*IL-22*, *Reg3g*, *S100a9*) in colon from WT and $Villin^{Cre}Ahr^{fl/fl}$ mice at day 7.

(E) *C. rodentium* burdens in colon, liver, and spleen. Bars show the median and each symbol represents an individual mouse.

(F) Colon sections stained for E-cadherin (green), *C. rodentium* (red), and DAPI (blue). Scale bars, 50 μm.

(G) Survival plot of mice infected with *C. rodentium* (WT, n = 7; $Villin^{Cre}Ahr^{fl/fl}$, n = 7).

(H) qPCR analysis of the goblet cell marker *Muc2* and enterocyte marker *Car4* (WT, n = 7; $Villin^{Cre}Ahr^{fl/fl}$, n = 7).

(I) Representative image of AB-PAS staining in WT and $Villin^{Cre}Ahr^{fl/fl}$. Scale bars, 50 μm.

(J) Quantification of the number of AB-PAS-positive cells per 20 crypts in WT and $Villin^{Cre}Ahr^{fl/fl}$.

Error bars, mean + SEM. *$p < 0.05$, **$p < 0.01$, ***$p < 0.001$, ****$p < 0.0001$ as calculated by one-way ANOVA with Tukey post-test.

the ability of intestinal stem cells to repair and differentiate in response to tissue damage, leading to profound effects on resistance to infection and formation of colorectal cancer. These defects could be repaired by exposure to dietary AHR ligands in $Villin^{Cre}R26^{LSL\text{-}Cyp1a1}$ mice, which have an intact *Ahr*, whereas $Villin^{Cre}Ahr^{fl/fl}$ mice lacking *Ahr* in IECs could not be rescued.

Thus, AHR fulfils a critical role in intestinal stem cells by calibrating their response to Wnt-β-catenin signals, thereby allowing coordinated stem cell renewal and differentiation.

RESULTS

AHR Promotes Barrier Function through Direct Activity on IECs

Given the profound impact of *Ahr* deficiency on intestinal homeostasis, we set out to define whether *Ahr* deficiency in hematopoietic versus non-hematopoietic cells affects mice differently during infection with the intestinal pathogen *Citrobacter rodentium*. For this purpose, we set up bone marrow chimeras, transferring bone marrow either from *Ahr*-deficient donors into wild-type hosts ($Ahr^{-/-}$ → WT) or from wild-type B6 donors into *Ahr*-deficient hosts (WT → $Ahr^{-/-}$). Although both types of chimeras eventually succumbed to infection, mice with *Ahr* deficiency in the non-hematopoietic compartment exhibited accelerated mortality (Figure 1A), suggesting that AHR function is particularly important in IECs. We subsequently crossed mice with a floxed *Ahr* locus to *Villin-Cre* mice to restrict *Ahr* deficiency to IECs (referred to as $Villin^{Cre}Ahr^{fl/fl}$). These mice had an intact immune response to *C. rodentium* infection, with similar numbers of colonic ILC3 and Th17 cells as WT mice (Figure 1B) and comparable or even enhanced expression of IL-22 and its target genes *Reg3g* and *S100a9* (Figures 1C and 1D). Nevertheless, infection of $Villin^{Cre}Ahr^{fl/fl}$ mice with *C. rodentium* led to deep penetration of bacteria to the intestinal crypts, bacterial dissemination to the liver and spleen (Figures 1E and 1F), and rapid onset of mortality (Figure 1G). This indicates that AHR activation in immune cells is not sufficient to protect against *C. rodentium* infection and that AHR signaling in IECs serves a cell-autonomous role in promoting epithelial barrier function in an IL-22-independent manner.

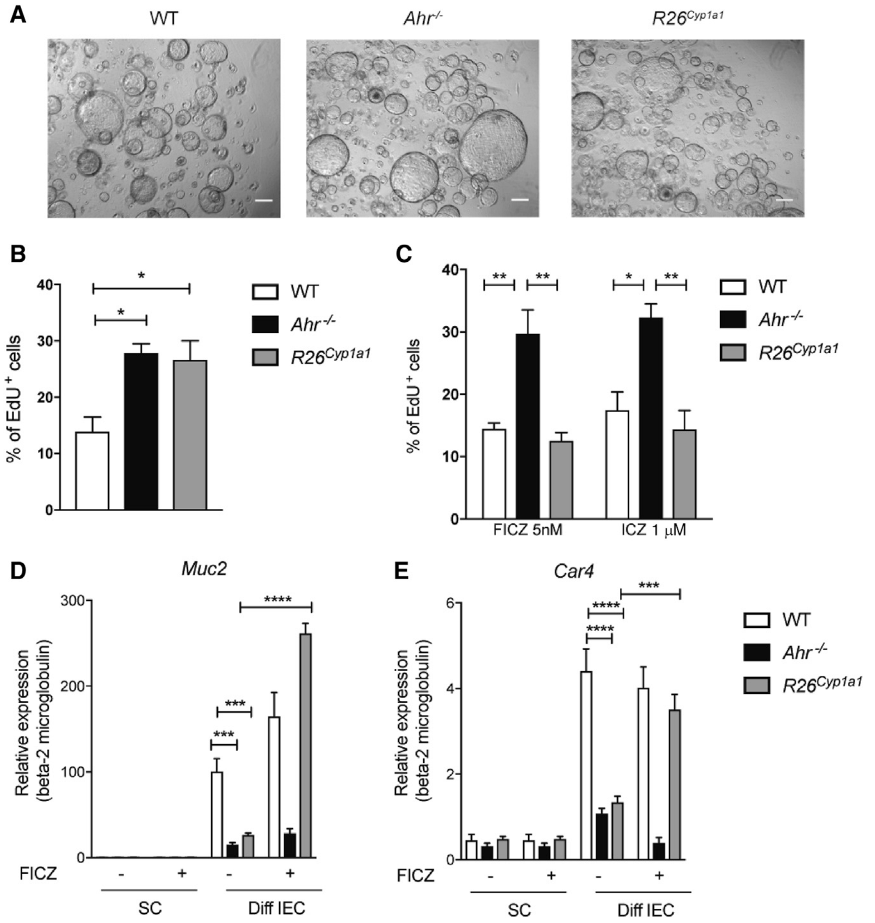

Figure 2. AHR Pathway Is Required for Epithelial Cell Differentiation from Crypt Stem Cells

(A) Images of stem cell (SC) organoids from WT, *Ahr*$^{-/-}$, and *R26*^{Cyp1a1} mice. Scale bars, 50 μm.

(B) Percentage of EdU^{+} cells in SC organoid cultures.

(C) Percentage of EdU^{+} cells in SC organoid cultures treated with 5 nM FICZ or 1 μM ICZ.

(D and E) qPCR analysis of the goblet cell marker *Muc2* and enterocyte marker *Car4* in SC or differentiated (diff) organoids treated or not with 5 nM FICZ for 4 days.

Data represent pooled results of at least two independent experiments (n = 6). Error bars, mean + SEM. *p < 0.05, **p < 0.01, ***p < 0.001, ****p < 0.0001 as calculated by one-way and two-way ANOVA with Tukey post-test.

C. rodentium is an attaching effacing pathogen that causes IEC apoptosis (Vallance et al., 2003) and necessitates replenishment of damaged IECs for maintenance of barrier integrity and repair processes. Resistance to *C. rodentium* infection varies between different strains of mice and susceptible strains are characterized by aberrant R-spondin 2 (RSPO2)-mediated Wnt-β-catenin activation which causes excessive ISC proliferation and poor differentiation into epithelial subtypes (Papapietro et al., 2013). Compared with *Ahr*$^{fl/fl}$ mice, infected *Villin*^{Cre}*Ahr*$^{fl/fl}$ mice had significantly lower expression of *Muc2* and *Car4* (Figure 1H), with corresponding reduction of goblet cells (Figures 1I and 1J). This indicates a defective repair process following infection, which is likely to contribute to the severe barrier defect that leads to dissemination of bacteria in this strain.

AHR Dysregulation in IECs Interferes with Regulation of Wnt-β-Catenin Signaling

The intestinal epithelium continuously renews itself from crypt stem cells that differentiate into short-lived specialized epithelial subtypes (Sato et al., 2009). This is a highly regulated process that depends on orchestrated Wnt-β-catenin signaling in crypt stem cells. In order to directly investigate the role of AHR in the process of crypt stem cell proliferation and differentiation, we generated colon organoids (Sato and Clevers, 2013). Comparing organoids from wild-type mice with mice exhibiting dysregulated AHR signaling (*R26*^{Cyp1a1}) and mice lacking *Ahr* (*Ahr*$^{-/-}$) allowed us to investigate the role of AHR pathway activation in stem cells. Colon organoids from *Ahr*$^{-/-}$ or *R26*^{Cyp1a1} crypts showed increased proliferation, as indicated by higher uptake of EdU (Figures 2A and 2B). The proliferative response was normalized in *R26*^{Cyp1a1} but not *Ahr*$^{-/-}$ organoids by addition of AHR ligands FICZ (6-formylindolo[3,2-*b*]carbazole) or ICZ (indolo[3,2-*b*]carbazole) into the culture medium (Figure 2C). As expected, withdrawal of WNT in wild-type stem cell organoid cultures led to an increase in expression of the goblet cell marker *Muc2* (Figure 2D, left, white bars) and enterocyte marker *Car4* (Figure 2E, right, white bars). However, organoids with dysregulated AHR (Figures 2D and 2E, black and gray bars) had substantially lower expression of *Muc2* and *Car4*, indicating compromised differentiation to goblet and enterocyte fate. Nevertheless, addition of AHR ligands restored goblet cell and enterocyte differentiation in organoids derived from *R26*^{Cyp1a1} (gray bars) but not of *Ahr*$^{-/-}$ (black bars) mice to wild-type levels. This indicates that appropriate stimulation of the AHR pathway is required for epithelial cell differentiation from crypt stem cells.

Inflammation-Induced Tumorigenesis Is Enhanced in Mice with *Ahr*-Deficient Epithelium

Thus far, our data indicate that dysregulated AHR in IECs leads to aberrant inflammation and enhanced stem cell proliferation. To determine whether this phenotype is apparent under steady-state conditions, we compared stem cell proliferation, epithelial cell differentiation, and basic inflammatory tone in untreated *Villin*^{Cre}*Ahr*$^{fl/fl}$ and *Villin*^{Cre}*R26*$^{LSL\text{-}Cyp1a1}$ mice.

For this we made use of a mouse strain in which LGR5^{+} crypt stem cells are marked with GFP, *Lgr5*$^{Egfp\text{-}Ires\text{-}creErt2}$ (Barker et al., 2007).

Proliferation of LGR5^{+} stem cells, measured by Ki67 expression, was already significantly increased in young *Ahr*-deficient mice compared to littermate controls (Figure 3A). However, a numeric increase in LGR5^{+} stem cells manifested only in older

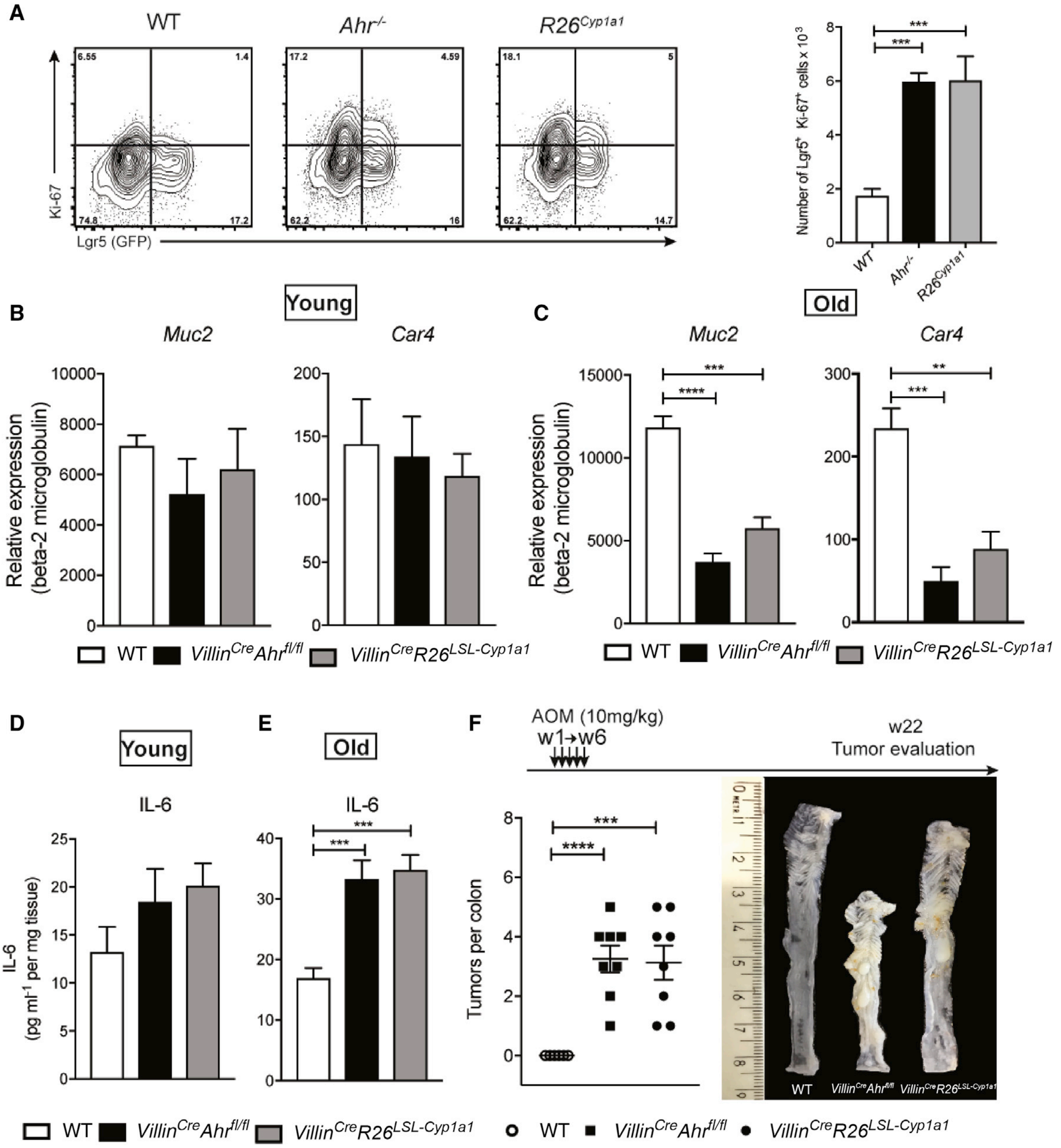

Figure 3. Dysregulated *Ahr* in IECs Leads to Enhanced Stem Cell Proliferation and Increased Inflammation

(A) Flow cytometry analysis of Lgr5 and Ki-67 expression in EpCam$^+$CD45$^-$ cells and absolute number of Lgr5$^+$Ki-67$^+$ cells at steady state from 5- to 8-week-old mice.

(B and C) qPCR analysis of the goblet cell marker *Muc2* and enterocyte marker *Car4* from sorted EpCam$^+$ cells (WT, n = 6; *Villin^{Cre}Ahr$^{fl/fl}$*, n = 6; *Villin^{Cre}R26$^{LSL-Cyp1a1}$*, n = 6) in young (5 to 9 weeks) and old (14 to 16 weeks) mice.

(D and E) IL-6 protein content in colon explant cultures at steady state (WT, n = 6; *Villin^{Cre}Ahr$^{fl/fl}$*, n = 6; *Villin^{Cre}R26$^{LSL-Cyp1a1}$*, n = 6) from young and old mice.

(F) Number of colon tumors in WT (n = 8), *Villin^{Cre}Ahr$^{fl/fl}$* (n = 8), and *Villin^{Cre}R26$^{LSL-Cyp1a1}$* (n = 8) mice injected with 10 mg/kg of azoxymethane once a week for 6 weeks. Representative image of colon in mice 22 weeks after the first azoxymethane injection.

Error bars, mean + SEM., **p < 0.01, ***p < 0.001, ****p < 0.0001 as calculated by one-way ANOVA with Tukey post-test. See also Figures S1A and S1B.

mice with a compromised AHR pathway (Figure S1). Since we were not able to maintain *Ahr*-deficient *Lgr5$^{Egfp-Ires-creErt2}$* reporter mice viable for long enough, we used mice heterozygous for *Ahr* deletion, which were partially affected. With respect to epithelial differentiation, we did not observe any significant difference in young mice (aged between 5 and 8 weeks) (Figure 3B), while *Muc2* and *Car4* expression were strongly reduced in older mice aged between 14 and 16 weeks (Figure 3C). Likewise, IL-6 levels were not different at steady state in young mice, but were elevated in older mice (Figures 3D and 3E).

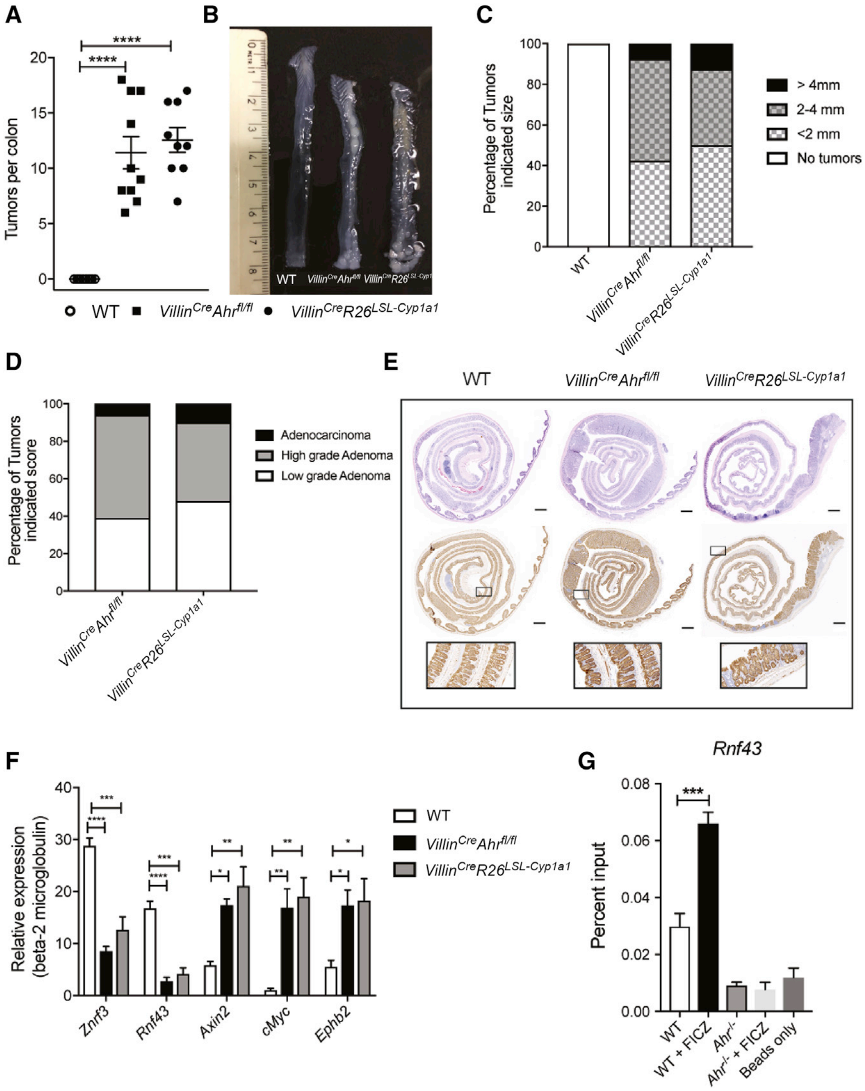

Figure 4. Inflammation-Induced Tumorigenesis in Mice with *Ahr*-Deficient Epithelium

(A) Number of colon tumors in WT (n = 10), *Villin^{Cre}Ahr$^{fl/fl}$* (n = 10), and *Villin^{Cre}R26$^{LSL-Cyp1a1}$* (n = 9) mice.

(B) Representative image of colon after AOM/DSS treatment.

(C) Size of colon tumors in WT (n = 10), *Villin^{Cre}Ahr$^{fl/fl}$* (n = 10), and *Villin^{Cre}R26$^{LSL-Cyp1a1}$* (n = 9) mice.

(D) Scoring of colon tumors *Villin^{Cre}Ahr$^{fl/fl}$* (n = 10) and *Villin^{Cre}R26$^{LSL-Cyp1a1}$* (n = 9) mice.

(E) Representative images of hematoxylin and eosin (H&E) (top) and β-catenin staining of whole intestine (middle) and focus (bottom) of the indicated mice 60 days after injection of azoxymethane. Scale bars, 50 μm.

(F) qPCR analysis of Wnt-negative regulators (*Znrf3* and *Rnf43*) and WNT target genes (*Axin2*, *cMyc*, and *Ephb2*) in the colon (WT, n = 5; *Villin^{Cre}Ahr$^{fl/fl}$*, n = 5; *Villin^{Cre}R26$^{LSL-Cyp1a1}$*, n = 5) after AOM/DSS treatment.

(G) Chromatin immunoprecipitation (ChIP) analysis of AHR interaction with *Rnf43* promoter from lanima propria of WT, WT treated with FICZ, *Ahr$^{-/-}$*, and *Ahr$^{-/-}$* treated with FICZ.

Error bars, mean + SEM. *$p < 0.05$, **$p < 0.01$, ***$p < 0.001$, ****$p < 0.0001$ as calculated by one-way and two-way ANOVA with Tukey post-test. See also Figures S2A–S2D.

The combination of enhanced stem cell proliferation and subclinical inflammation is commonly associated with malignant transformation and colorectal cancer. Indeed, *Villin^{Cre}Ahr$^{fl/fl}$* and *Villin^{Cre}R26$^{LSL-Cyp1a1}$* mice, when exposed to the mutagen azoxymethane (AOM), developed large tumors within 4 months of AOM application, whereas no tumors were observed in wild-type mice (Figure 3F). In order to accelerate the process of tumorigenesis, we applied the widely used AOM/DSS (dextran sulfate sodium) model in which one dose of AOM was followed by two cycles of DSS. As *Ahr*-deficient mice are highly sensitive to DSS (Furumatsu et al., 2011), we had to reduce the dose to 1% DSS, which is suboptimal for wild-type mice. Consequently, no tumors were observed in wild-type mice under this treatment regime, while both *Villin^{Cre}Ahr$^{fl/fl}$* and *Villin^{Cre}R26$^{LSL-Cyp1a1}$* mice developed numerous tumors throughout the colon (Figures 4A–4C, top row), ranging in severity from low-grade adenoma to adenocarcinoma (Figure 4D). In agreement with published data on increased levels of β-catenin in *Ahr$^{-/-}$* mice (Kawajiri et al., 2009), both strains of mice with dysregulated AHR in IECs had substantially increased β-catenin expression in the intestine (Figure 4E, bottom row). We next investigated the underlying mechanism for Wnt-β-catenin pathway dysregulation in our mice. We focused on the tumor suppressors RNF43 and ZNRF3, which are transmembrane E3 ubiquitin ligases expressed in LGR5^{+} stem cells that regulate WNT signals by targeting WNT receptors for degradation in an R-spondin-sensitive manner (Hao et al., 2012; Koo et al., 2012; Tsukiyama et al., 2015). Both *Rnf43* and *Znrf3* were expressed at significantly lower levels in epithelium from *Villin^{Cre}Ahr$^{fl/fl}$* and *Villin^{Cre}R26$^{LSL-Cyp1a1}$* mice, while WNT target genes such as *Axin2*, *cMyc*, and *Ephb2* were expressed at higher levels in steady-state conditions (Figure S1A) and following AOM/DSS administration (Figure 4F). ChIP qPCR of intestinal epithelial cells established *Rnf43* as a direct target of AHR, suggesting that this tumor suppressor, which acts in a complex with ZNRF3, is transcriptionally regulated by AHR (Figure 4G). Similar results were obtained in colon organoids (Figure S2B). Thus, dysregulated feedback signaling in the Wnt-β-catenin pathway causes increased expansion of crypt stem cells in mice with compromised physiological AHR signaling. In further corroboration of defective feedback regulation, organoids from *Ahr*-deficient strains grew and survived in the absence of R-Spondin1, as shown for *Rnf45/Znrf3* mutants (Koo et al., 2012, 2015), whereas wild-type organoids died upon withdrawal of R-spondin1 (Figure S2C).

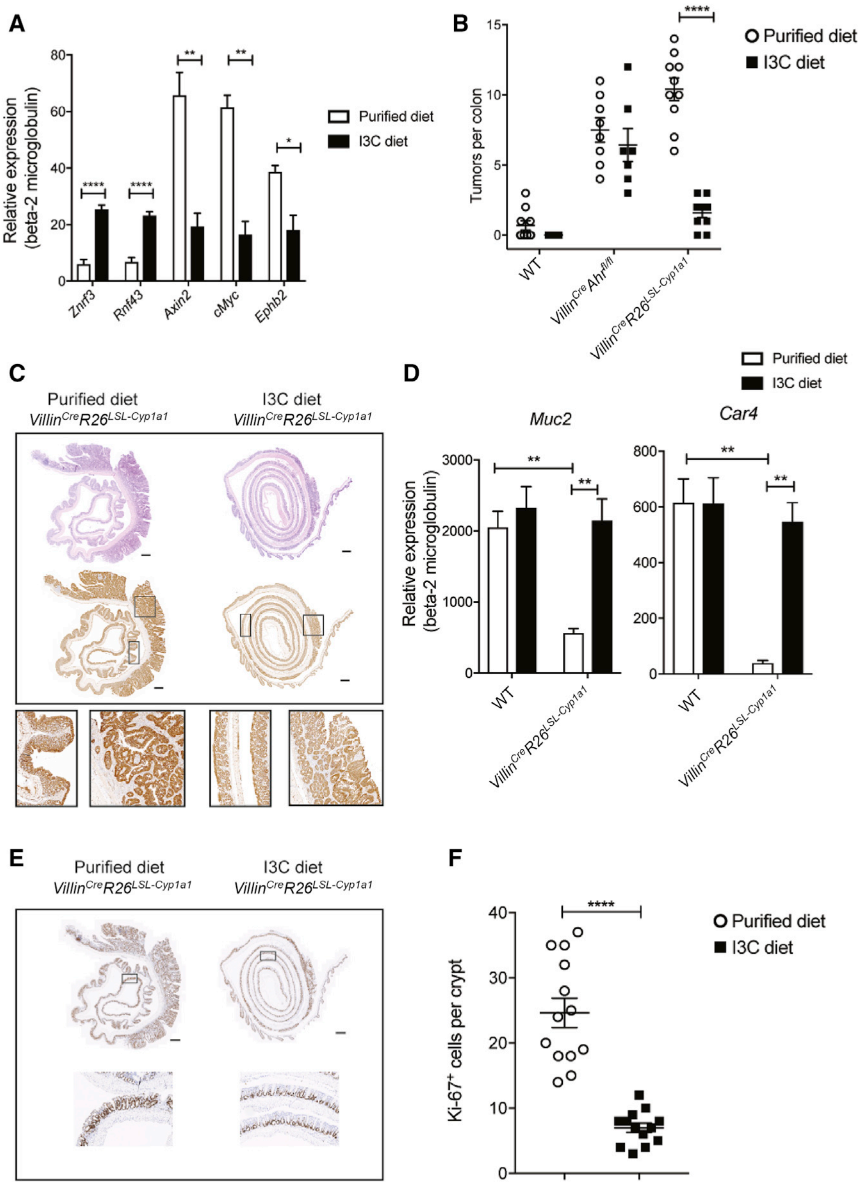

Figure 5. Dietary AHR Ligands Prevent Tumorigenesis and Restore Regulation of the WNT Pathway

(A) qPCR analysis of WNT negative regulators (*Znrf3* and *Rnf43*) and WNT target genes (*Axin2*, *cMyc*, and *Ephb2*) in the colon of *Villin*^{Cre}*R26*$^{LSL\text{-}Cyp1a1}$ mice fed purified or I3C diet (n = 5).

(B) Number of colon tumors in WT (n = 10), *Villin*^{Cre}*Ahr*$^{fl/fl}$, and *Villin*^{Cre}*R26*$^{LSL\text{-}Cyp1a1}$ (n = 10), fed purified or I3C diet after AOM/DSS treatment.

(C) Representative images of hematoxylin and eosin (H&E) and β-catenin of colon tumors in *Villin*^{Cre}*R26*$^{LSL\text{-}Cyp1a1}$ fed purified or I3C diet. Scale bars, 50 μm.

(D) qPCR analysis of *Muc2* and *Car4* in colon from WT (n = 5) and *Villin*^{Cre}*R26*$^{LSL\text{-}Cyp1a1}$ (n = 5), fed purified or I3C diet.

(E) Representative images of Ki-67 of colon tumors in *Villin*^{Cre}*R26*$^{LSL\text{-}Cyp1a1}$ mice fed purified or I3C diet. Scale bars, 50 μm.

(F) Quantification of the number of Ki-67^{+} cells in *Villin*^{Cre}*R26*$^{LSL\text{-}Cyp1a1}$ mice fed purified or I3C diet.

Error bars, mean + SEM. *p < 0.05, **p < 0.01, ****p < 0.0001, as calculated by unpaired t test and two-way ANOVA with Tukey post-test. See also Figures S3A and S3B.

Dietary AHR Ligands Prevent Tumorigenesis and Restore Regulation of Wnt-β-Catenin Pathway

The notion that a diet enriched in green vegetables reduces the risk of cancer formation is widely distributed, although scientific evidence from several clinical trials remains inconsistent (Mayne et al., 2016; Potter and Steinmetz, 1996; Terry et al., 2001). In particular, there is no consensus for a molecular mechanism explaining the phenomenon of an inverse relationship between plant-derived food intake and cancer development. Some of the strongest endogenous AHR ligands are derived from phytochemicals such as indole-3-carbinol (I3C) that is converted to the high-affinity AHR ligand ICZ by exposure to stomach acid (Bjeldanes et al., 1991; Kleman et al., 1994). We previously showed that dietary application of I3C could cure the extreme sensitivity to enteric infection of *Villin*^{Cre}*R26*$^{LSL\text{-}Cyp1a1}$ mice (Schiering et al., 2017).

Here we fed *Villin*^{Cre}*R26*$^{LSL\text{-}Cyp1a1}$ mice with a purified control diet or purified diet supplemented with I3C for 2 weeks prior to as well as during treatment with AOM/DSS. Exposure to I3C diet for 2 weeks prior to AOM/DSS treatment sufficed to correct the expression of *Znrf3* and *Rnf43* (Figure 5A) in an AHR-dependent manner, as mice lacking *Ahr* in IEC did not upregulate these tumor suppressors (Figure S3B). Importantly, wild-type mice also expressed increased levels of *Znrf3* and *Rnf43* when exposed to I3C diet compared to control diet (Figure S3A). In line with reinstatement of negative feedback control of Wnt-β-catenin signaling by enhanced expression of *Znrf3* and *Rnf43* upon I3C diet exposure, we observed decreased expression of WNT target genes *Axin2*, *cMyc*, and *Ephb2* in *Villin*^{Cre}*R26*$^{LSL\text{-}Cyp1a1}$ mice. Wild-type littermate control mice fed with control purified diet showed a low level of tumor burden (Figure 5B) in contrast to what we had seen on our standard chow diet where wild-type mice never developed tumors (Figure 4A). This probably reflects the reduced availability of AHR ligands in the purified control diet as compared to standard chow diet. Strikingly, addition of I3C to purified diet reduced tumor formation to levels seen in wild-type mice and decreased β-catenin expression in the intestine and the tumor (Figure 5C). This effect was dependent on AHR activation in IECs as I3C diet did not reduce tumor formation in *Villin*^{Cre}*Ahr*$^{fl/fl}$ mice (Figure 5B). Furthermore, dietary substitution with AHR ligands restored the defect in epithelial cell differentiation (Figure 5D) and reduced the hyperproliferation of crypt stem cells

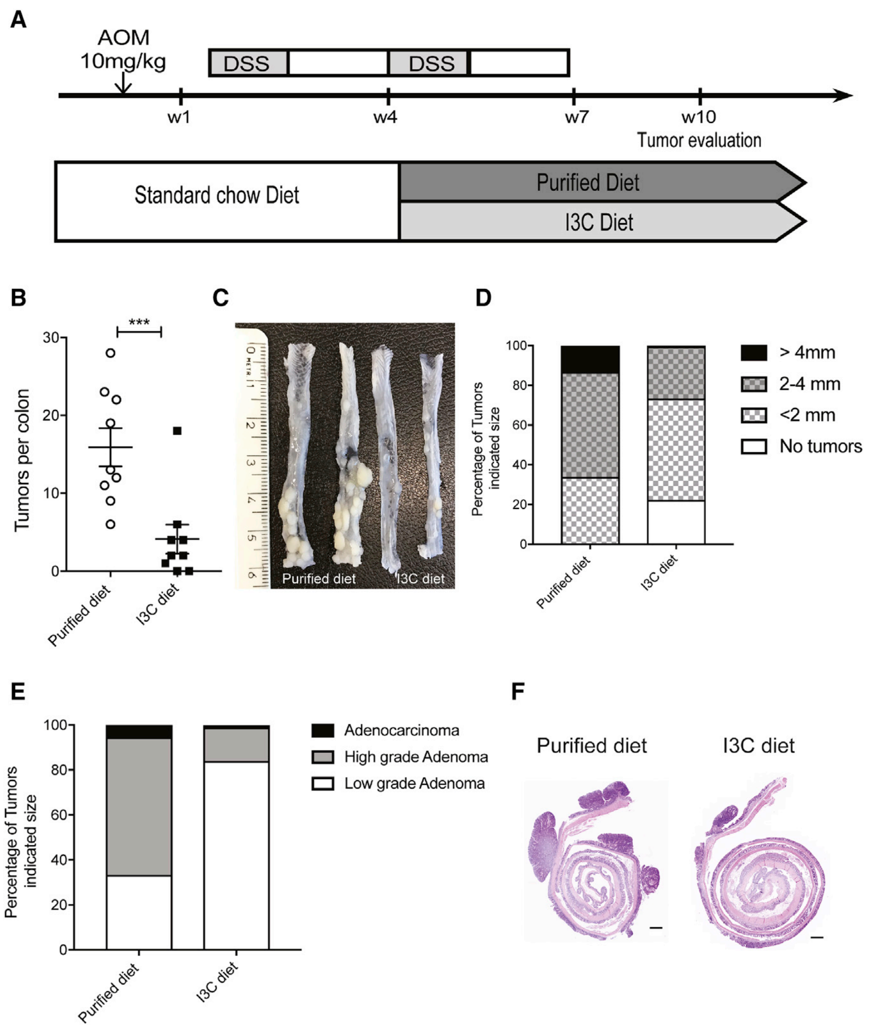

Figure 6. Dietary AHR Ligands Can Halt Progression of Tumorigenesis

(A) *Villin*^{Cre}*R26*$^{LSL\text{-}Cyp1a1}$ were injected with 10 mg/kg of AOM followed with one cycle of 1% DSS on standard chow diet. For the second cycle of DSS, mice were put either on a purified diet or I3C diet until the end of the experiment.

(B) Number of colon tumors in WT (n = 10), *Villin*^{Cre} *Ahr*$^{fl/fl}$, and *Villin*^{Cre}*R26*$^{LSL\text{-}Cyp1a1}$ (n = 10) mice, fed purified or I3C diet after AOM/DSS treatment.

(C) Representative image of colon after AOM/DSS treatment.

(D and E) Size (D) and score (E) of tumors in *Villin*^{Cre}*R26*$^{LSL\text{-}Cyp1a1}$ mice fed purified or I3C diet.

(F) Representative images of hematoxylin and eosin (H&E) colon tumors in *Villin*^{Cre}*R26*$^{LSL\text{-}Cyp1a1}$ mice fed purified or I3C diet. Scale bars, 50 μm.

Error bars, mean + SEM. ***$p < 0.001$, as calculated by paired t test.

(Figure 5E) in line with our findings in organoid cultures (Figures 2B and 2C).

Thus, normalization of AHR signaling in response to dietary or natural ligands restored the regulation of the Wnt-β-catenin pathway, allowing repair of DSS-induced tissue damage by supporting epithelial cell differentiation and protection from inflammation-induced tumorigenesis.

Dietary AHR Ligands Can Halt Progression of Tumorigenesis

We next investigated whether dietary supplementation with AHR ligands after the onset of tumorigenesis affects tumor progression. *Villin*^{Cre}*R26*$^{LSL\text{-}Cyp1a1}$ mice were subjected to AOM followed by DSS on standard chow diet. Four weeks later when four out of five mice had developed at least one tumor (data not shown), mice received the second dose of DSS and were placed on either purified control diet or purified diet supplemented with I3C (Figure 6A); tumors were assessed 6 weeks later. Whereas all mice on the purified control diet developed tumors (Figures 6B and 6C), two out of nine mice on the I3C diet did not develop tumors (Figure 6D) and the remaining mice had reduced numbers of tumors—mainly low-grade adenomas (Figure 6E). Thus, normalization of AHR signaling even after tumor induction has beneficial effects in reducing tumor load and severity, indicating the therapeutic potential of dietary AHR ligand I3C.

DISCUSSION

AHR is widely expressed in the intestine and absence of AHR has been shown to result in loss of several immune cell types or their functional activities (Kiss et al., 2011; Li et al., 2011; Qiu et al., 2012). However, as we showed here, *Ahr* deficiency profoundly affected intestinal homeostasis through direct activity on intestinal epithelial cells. Our previous research established an important role of the AHR pathway in IECs as "gatekeeper" for the supply of ligands to intestinal immune cells (Schiering et al., 2017). However, we now found additional IEC-intrinsic AHR functions that did not involve mucosal immune cells such as ILC3 or Th17 cells and were independent of their production of IL-22. This was borne out of experiments with mice lacking the AHR pathway in IECs (*Villin*^{Cre}*Ahr*$^{fl/fl}$), which have a normal intestinal immune compartment and no impairment in IL-22 production, but nevertheless were as susceptible to infection with *C. rodentium* as mice with overactive CYP1A1 in IECs (*Villin*^{Cre}*R26*$^{LSL\text{-}Cyp1a1}$). Instead, we identified an important role for AHR in controlling the response of crypt stem cells to WNT signals that affect proliferation and the differentiation of epithelial subsets. Repair of damaged epithelium is a crucial feature for resistance to intestinal infection with pathogens that cause epithelial damage, such as *C. rodentium* and replacement of mucus-producing goblet cells is vital in order to prevent dissemination of bacteria. The failure to achieve this in a timely manner was clearly evident in *Villin*^{Cre}*Ahr*$^{fl/fl}$ or *Villin*^{Cre}*R26*$^{LSL\text{-}Cyp1a1}$ mice that both succumbed to severe bacterial dissemination and were also highly susceptible to chemically induced epithelial damage by DSS. A previous publication

demonstrated a link between susceptibility to *C. rodentium* infection and RSPO2, a secreted G-coupled receptor that enhances Wnt-β-catenin signaling (Papapietro et al., 2013), but the underlying mechanisms that regulate WNT signaling in susceptible strains remained uncharacterized. Interestingly, mouse strains that are susceptible to *C. rodentium*, such as FVB and AKR, express the low-affinity Ahr^{d} allele as do mice with a floxed *Ahr* locus (Walisser et al., 2005) and these are substantially more affected by *C. rodentium* infection than C57BL/6 mice with the high-affinity Ahr^{b} allele (data not shown).

Aberrant stem cell proliferation was evident at steady state in older mice with a dysregulated AHR pathway, suggesting that the intestinal barrier is compromised to some extent prior to any challenge. This would explain why such mice expressed elevated levels of IL-6 that can promote malignant progression in colon cancer (West et al., 2015). IL-22 that has also been linked to colon cancer in mice and humans (Jiang et al., 2013; Kryczek et al., 2014) is elevated in $Villin^{Cre}Ahr^{fl/fl}$ mice, probably due to increased levels of AHR ligands accessible to mucosal immune cells, as *Ahr*-deficient IECs do not express CYP1A1. However, this cytokine is profoundly depleted in $Villin^{Cre}R26^{LSL\text{-}Cyp1a1}$ mice (Schiering et al., 2017) yet they are similarly prone to tumorigenesis as $Villin^{Cre}Ahr^{fl/fl}$ mice. Thus, IL-6 rather than IL-22 is likely to provide the inflammatory setting in these mice.

The progression from inflammation to tumorigenesis is well established (Schäfer and Werner, 2008) and our mice readily developed mixed colon adenomas/adenocarcinomas in the standard AOM/DSS model in line with previous data in global $Ahr^{-/-}$ mice (Díaz-Díaz et al., 2016) or even with the mutagen AOM alone. *In vitro* studies on cell lines proposed that AHR functions as part of an E3 ubiquitin ligase complex that serves to ubiquinate β-catenin and target it for degradation (Luecke-Johansson et al., 2017; Ohtake et al., 2007). Our results provide evidence that Wnt-β-catenin pathway dysregulation in *Ahr*-deficient IECs occurred upstream of β-catenin degradation, affecting the response to WNT signals themselves through the expression of the two related E3 ubiquitin ligases (ZNRF3 and RNF43) that degrade the Wnt frizzled receptors (Koo et al., 2012) in $LGR5^{+}$ stem cells. It is conceivable that the E3 ubiquitin ligase activity of AHR contributed to β-catenin accumulation in our study. However, we observed a selective defect in induction of *Znrf3* and *Rnf43* in IECs with dysregulated AHR, while expression of other WNT pathway targets such as *Axin2*, *cMyc*, and *Ephb2* was enhanced. Thus, our findings strongly support a model by which AHR modulates β-catenin levels through transcriptional induction of negative regulators of the Wnt-β-catenin pathway. Our data further support the notion that WNT responsiveness of intestinal stem cells is normally restrained by physiological AHR signals, and restoration of the AHR response in $Villin^{Cre}R26^{LSL\text{-}Cyp1a1}$ mice via dietary supplementation reset the feedback control of Wnt-β-catenin signaling in crypt stem cells and promoted their differentiation, resulting in protection from tumorigenesis.

The impact of nutrition on tumorigenesis is widely documented (Zitvogel et al., 2017) and in particular a diet high in fat was proposed to augment intestinal stem cell numbers and—in the context of Apc^{Min} mutation—promote susceptibility to cancerogenesis (Beyaz et al., 2016). Conversely, a diet rich in vegetables and phytochemicals is thought to be beneficial. It remains to be investigated whether experimental high-fat diets contain similar levels of phytochemicals generating AHR ligands as control diets and it might be worth considering that the deleterious effects of high-fat diet on cancerogenesis could have been partly due to lack of phytochemicals rather than increased fat content.

We found here that the purified control diet, which is used as comparison for I3C supplemented diet, appeared to contain reduced levels of AHR ligands and as a consequence produced some features of *Ahr* deficiency in wild-type mice. As an example, wild-type mice fed purified control diet developed some tumors within 10 weeks of AOM/DSS, whereas this was not observed when they were fed the standard chow diet.

There are numerous reports on I3C as a cancer-preventive agent (Díaz-Díaz et al., 2016; Kim and Milner, 2005; Weng et al., 2008). However, consensus on the mode of action and mechanistic details of how nutrition interfaces with the inflammatory process are missing. We have provided evidence that supplementation with dietary I3C to $Villin^{Cre}R26^{LSL\text{-}Cyp1a1}$ mice corrected the expression of *Rnf43* and *Znrf3* in an AHR-dependent manner, thus normalizing the negative feedback regulation of the Wnt-β-catenin pathway, which resulted in significantly reduced or absent tumorigenesis. Furthermore, dietary supplementation with AHR ligands in mice with a dysregulated AHR pathway normalized crypt stem cell hyperproliferation and the differentiation process to goblet cells and enterocytes. Dietary supplementation with AHR ligands also worked in a therapeutic setting after onset of tumorigenesis, resulting in significantly reduced numbers of tumors with a more benign phenotype.

A number of recent studies have focused on the provision of AHR ligands by the microbiota (Hubbard et al., 2015; Lamas et al., 2016; Zelante et al., 2013), while our data emphasize dietary components as major contributors to AHR stimulation. Microbiota and diet are interlinked and it remains to be determined whether a diet promoting physiological AHR activation influences the growth of tryptophan-metabolizing bacteria that may generate AHR ligands. As environmental triggers via AHR are involved in transcriptional regulation of a critical WNT feedback regulator in intestinal stem cells, dietary supplementation with AHR ligands may positively influence crucial pathways involved in epithelial cell differentiation and regulation of inflammatory processes.

STAR★METHODS

Detailed methods are provided in the online version of this paper and include the following:

- KEY RESOURCES TABLE
- CONTACT FOR REAGENT AND RESOURCE SHARING
- EXPERIMENTAL MODEL AND SUBJECT DETAILS
- METHOD DETAILS
  - Generation of BM chimeras
  - Infection with Citrobacter rodentium
  - Isolation of lamina propria cells and flow cytometry
  - Colon explant cultures
  - Immunohistochemistry and Immunofluorescence Staining

- ○ Quantitative Real-time Polymerase Chain Reaction
- ○ Chromatin immunoprecipitation
- ○ Azoxymethane–DSS model of colorectal tumorigenesis
- ○ Organoid Culture
- ○ EdU Click-iT cell cycle flow cytometry assay
- ○ Dietary intervention
- ● QUANTIFICATION AND STATISTICAL ANALYSIS

SUPPLEMENTAL INFORMATION

Supplemental Information includes three figures and can be found with this article online at https://doi.org/10.1016/j.immuni.2018.07.010.

ACKNOWLEDGMENTS

This work was supported by the Francis Crick Institute which receives its core funding from Cancer Research UK (FC001159 and FC001105), The UK Medical Research Council (FC001159 and FC001105), and the Wellcome Trust (FC001159 and FC001105). We would like to acknowledge the Biological Research Facility at the Francis Crick Institute for expert breeding and maintenance of our mouse strains, the Histopathology Facility for help with sections and staining, and the Flow Cytometry Facility and Cell Services for provision of organoid culture supernatants. We are grateful to Dr. Gad Frankel for rabbit antiserum against *Citrobacter rodentium* and for *Citrobacter* strain ICC169. The study was supported by a Wellcome Advanced Investigator Grant (100910/Z/13/Z) and a CRUK cancer immunology project grant (19691) to B.S. and a Sir Henry Wellcome Fellowship (103156/B/13/Z) and a Fondation Acteria award to C.S.

AUTHOR CONTRIBUTIONS

A.M. designed, performed, and analyzed most of the experiments with input from S.O., S.C., and Y.L. E.N. performed the histology and E.R. provided the tumor scoring. M.R.M. carried out organoid survival experiments, V.L. provided advice and reagents for organoid cultures, and B.S. and C.S. conceived the project and wrote the manuscript.

DECLARATION OF INTERESTS

The authors declare no competing interests.

Received: January 8, 2018
Revised: March 23, 2018
Accepted: July 20, 2018
Published: August 14, 2018

REFERENCES

Andersson, P., McGuire, J., Rubio, C., Gradin, K., Whitelaw, M.L., Pettersson, S., Hanberg, A., and Poellinger, L. (2002). A constitutively active dioxin/aryl hydrocarbon receptor induces stomach tumors. Proc. Natl. Acad. Sci. USA *99*, 9990–9995.

Barker, N., van Es, J.H., Kuipers, J., Kujala, P., van den Born, M., Cozijnsen, M., Haegebarth, A., Korving, J., Begthel, H., Peters, P.J., and Clevers, H. (2007). Identification of stem cells in small intestine and colon by marker gene Lgr5. Nature *449*, 1003–1007.

Beyaz, S., Mana, M.D., Roper, J., Kedrin, D., Saadatpour, A., Hong, S.J., Bauer-Rowe, K.E., Xifaras, M.E., Akkad, A., Arias, E., et al. (2016). High-fat diet enhances stemness and tumorigenicity of intestinal progenitors. Nature *531*, 53–58.

Bjeldanes, L.F., Kim, J.Y., Grose, K.R., Bartholomew, J.C., and Bradfield, C.A. (1991). Aromatic hydrocarbon responsiveness-receptor agonists generated from indole-3-carbinol in vitro and in vivo: comparisons with 2,3,7,8-tetrachlorodibenzo-p-dioxin. Proc. Natl. Acad. Sci. USA *88*, 9543–9547.

Borten, M.A., Bajikar, S.S., Sasaki, N., Clevers, H., and Janes, K.A. (2018). Automated brightfield morphometry of 3D organoid populations by OrganoSeg. Sci. Rep. *8*, 5319.

Denison, M.S., and Nagy, S.R. (2003). Activation of the aryl hydrocarbon receptor by structurally diverse exogenous and endogenous chemicals. Annu. Rev. Pharmacol. Toxicol. *43*, 309–334.

Díaz-Díaz, C.J., Ronnekleiv-Kelly, S.M., Nukaya, M., Geiger, P.G., Balbo, S., Dator, R., Megna, B.W., Carney, P.R., Bradfield, C.A., and Kennedy, G.D. (2016). The aryl hydrocarbon receptor is a repressor of inflammation-associated colorectal tumorigenesis in mouse. Ann. Surg. *264*, 429–436.

Furumatsu, K., Nishiumi, S., Kawano, Y., Ooi, M., Yoshie, T., Shiomi, Y., Kutsumi, H., Ashida, H., Fujii-Kuriyama, Y., Azuma, T., and Yoshida, M. (2011). A role of the aryl hydrocarbon receptor in attenuation of colitis. Dig. Dis. Sci. *56*, 2532–2544.

Hao, H.X., Xie, Y., Zhang, Y., Charlat, O., Oster, E., Avello, M., Lei, H., Mickanin, C., Liu, D., Ruffner, H., et al. (2012). ZNRF3 promotes Wnt receptor turnover in an R-spondin-sensitive manner. Nature *485*, 195–200.

Hubbard, T.D., Murray, I.A., Bisson, W.H., Lahoti, T.S., Gowda, K., Amin, S.G., Patterson, A.D., and Perdew, G.H. (2015). Adaptation of the human aryl hydrocarbon receptor to sense microbiota-derived indoles. Sci. Rep. *5*, 12689.

Jiang, R., Wang, H., Deng, L., Hou, J., Shi, R., Yao, M., Gao, Y., Yao, A., Wang, X., Yu, L., and Sun, B. (2013). IL-22 is related to development of human colon cancer by activation of STAT3. BMC Cancer *13*, 59.

Kawajiri, K., Kobayashi, Y., Ohtake, F., Ikuta, T., Matsushima, Y., Mimura, J., Pettersson, S., Pollenz, R.S., Sakaki, T., Hirokawa, T., et al. (2009). Aryl hydrocarbon receptor suppresses intestinal carcinogenesis in ApcMin/+ mice with natural ligands. Proc. Natl. Acad. Sci. USA *106*, 13481–13486.

Kim, Y.S., and Milner, J.A. (2005). Targets for indole-3-carbinol in cancer prevention. J. Nutr. Biochem. *16*, 65–73.

Kiss, E.A., Vonarbourg, C., Kopfmann, S., Hobeika, E., Finke, D., Esser, C., and Diefenbach, A. (2011). Natural aryl hydrocarbon receptor ligands control organogenesis of intestinal lymphoid follicles. Science *334*, 1561–1565.

Kleman, M.I., Poellinger, L., and Gustafsson, J.A. (1994). Regulation of human dioxin receptor function by indolocarbazoles, receptor ligands of dietary origin. J. Biol. Chem. *269*, 5137–5144.

Koo, B.K., Spit, M., Jordens, I., Low, T.Y., Stange, D.E., van de Wetering, M., van Es, J.H., Mohammed, S., Heck, A.J., Maurice, M.M., and Clevers, H. (2012). Tumour suppressor RNF43 is a stem-cell E3 ligase that induces endocytosis of Wnt receptors. Nature *488*, 665–669.

Koo, B.K., van Es, J.H., van den Born, M., and Clevers, H. (2015). Porcupine inhibitor suppresses paracrine Wnt-driven growth of Rnf43;Znrf3-mutant neoplasia. Proc. Natl. Acad. Sci. USA *112*, 7548–7550.

Kryczek, I., Lin, Y., Nagarsheth, N., Peng, D., Zhao, L., Zhao, E., Vatan, L., Szeliga, W., Dou, Y., Owens, S., et al. (2014). IL-22(+)CD4(+) T cells promote colorectal cancer stemness via STAT3 transcription factor activation and induction of the methyltransferase DOT1L. Immunity *40*, 772–784.

Lamas, B., Richard, M.L., Leducq, V., Pham, H.P., Michel, M.L., Da Costa, G., Bridonneau, C., Jegou, S., Hoffmann, T.W., Natividad, J.M., et al. (2016). CARD9 impacts colitis by altering gut microbiota metabolism of tryptophan into aryl hydrocarbon receptor ligands. Nat. Med. *22*, 598–605.

Lee, J.S., Cella, M., McDonald, K.G., Garlanda, C., Kennedy, G.D., Nukaya, M., Mantovani, A., Kopan, R., Bradfield, C.A., Newberry, R.D., and Colonna, M. (2011). AHR drives the development of gut ILC22 cells and postnatal lymphoid tissues via pathways dependent on and independent of Notch. Nat. Immunol. *13*, 144–151.

Li, Y., Innocentin, S., Withers, D.R., Roberts, N.A., Gallagher, A.R., Grigorieva, E.F., Wilhelm, C., and Veldhoen, M. (2011). Exogenous stimuli maintain intraepithelial lymphocytes via aryl hydrocarbon receptor activation. Cell *147*, 629–640.

Liu, J.Z., van Sommeren, S., Huang, H., Ng, S.C., Alberts, R., Takahashi, A., Ripke, S., Lee, J.C., Jostins, L., Shah, T., et al.; International Multiple Sclerosis Genetics Consortium; International IBD Genetics Consortium (2015). Association analyses identify 38 susceptibility loci for inflammatory

bowel disease and highlight shared genetic risk across populations. Nat. Genet. *47*, 979–986.

Luecke-Johansson, S., Gralla, M., Rundqvist, H., Ho, J.C., Johnson, R.S., Gradin, K., and Poellinger, L. (2017). A molecular mechanism to switch the aryl hydrocarbon receptor from a transcription factor to an E3 ubiquitin ligase. Mol. Cell. Biol. *37*, 37.

Mayne, S.T., Playdon, M.C., and Rock, C.L. (2016). Diet, nutrition, and cancer: past, present and future. Nat. Rev. Clin. Oncol. *13*, 504–515.

McMillan, B.J., and Bradfield, C.A. (2007). The aryl hydrocarbon receptor sans xenobiotics: endogenous function in genetic model systems. Mol. Pharmacol. *72*, 487–498.

Mitchell, K.A., and Elferink, C.J. (2009). Timing is everything: consequences of transient and sustained AhR activity. Biochem. Pharmacol. *77*, 947–956.

Moura-Alves, P., Faé, K., Houthuys, E., Dorhoi, A., Kreuchwig, A., Furkert, J., Barison, N., Diehl, A., Munder, A., Constant, P., et al. (2014). AhR sensing of bacterial pigments regulates antibacterial defence. Nature *512*, 387–392.

Novellasdemunt, L., Antas, P., and Li, V.S. (2015). Targeting Wnt signaling in colorectal cancer. A review in the theme: cell signaling: proteins, pathways and mechanisms. Am. J. Physiol. Cell Physiol. *309*, C511–C521.

Ohtake, F., Baba, A., Takada, I., Okada, M., Iwasaki, K., Miki, H., Takahashi, S., Kouzmenko, A., Nohara, K., Chiba, T., et al. (2007). Dioxin receptor is a ligand-dependent E3 ubiquitin ligase. Nature *446*, 562–566.

Papapietro, O., Teatero, S., Thanabalasuriar, A., Yuki, K.E., Diez, E., Zhu, L., Kang, E., Dhillon, S., Muise, A.M., Durocher, Y., et al. (2013). R-spondin 2 signalling mediates susceptibility to fatal infectious diarrhoea. Nat. Commun. *4*, 1898.

Potter, J.D., and Steinmetz, K. (1996). Vegetables, fruit and phytoestrogens as preventive agents. IARC Sci. Publ. *139*, 61–90.

Qiu, J., Heller, J.J., Guo, X., Chen, Z.M., Fish, K., Fu, Y.X., and Zhou, L. (2012). The aryl hydrocarbon receptor regulates gut immunity through modulation of innate lymphoid cells. Immunity *36*, 92–104.

Sato, T., and Clevers, H. (2013). Growing self-organizing mini-guts from a single intestinal stem cell: mechanism and applications. Science *340*, 1190–1194.

Sato, T., Vries, R.G., Snippert, H.J., van de Wetering, M., Barker, N., Stange, D.E., van Es, J.H., Abo, A., Kujala, P., Peters, P.J., and Clevers, H. (2009). Single Lgr5 stem cells build crypt-villus structures in vitro without a mesenchymal niche. Nature *459*, 262–265.

Schäfer, M., and Werner, S. (2008). Cancer as an overhealing wound: an old hypothesis revisited. Nat. Rev. Mol. Cell Biol. *9*, 628–638.

Schiering, C., Wincent, E., Metidji, A., Iseppon, A., Li, Y., Potocnik, A.J., Omenetti, S., Henderson, C.J., Wolf, C.R., Nebert, D.W., and Stockinger, B. (2017). Feedback control of AHR signalling regulates intestinal immunity. Nature *542*, 242–245.

Schmidt, J.V., and Bradfield, C.A. (1996). Ah receptor signaling pathways. Annu. Rev. Cell Dev. Biol. *12*, 55–89.

Terry, P., Giovannucci, E., Michels, K.B., Bergkvist, L., Hansen, H., Holmberg, L., and Wolk, A. (2001). Fruit, vegetables, dietary fiber, and risk of colorectal cancer. J. Natl. Cancer Inst. *93*, 525–533.

Tsukiyama, T., Fukui, A., Terai, S., Fujioka, Y., Shinada, K., Takahashi, H., Yamaguchi, T.P., Ohba, Y., and Hatakeyama, S. (2015). Molecular role of RNF43 in canonical and noncanonical Wnt signaling. Mol. Cell. Biol. *35*, 2007–2023.

Vallance, B.A., Deng, W., Jacobson, K., and Finlay, B.B. (2003). Host susceptibility to the attaching and effacing bacterial pathogen *Citrobacter rodentium*. Infect. Immun. *71*, 3443–3453.

Walisser, J.A., Glover, E., Pande, K., Liss, A.L., and Bradfield, C.A. (2005). Aryl hydrocarbon receptor-dependent liver development and hepatotoxicity are mediated by different cell types. Proc. Natl. Acad. Sci. USA *102*, 17858–17863.

Weng, J.R., Tsai, C.H., Kulp, S.K., and Chen, C.S. (2008). Indole-3-carbinol as a chemopreventive and anti-cancer agent. Cancer Lett. *262*, 153–163.

West, N.R., McCuaig, S., Franchini, F., and Powrie, F. (2015). Emerging cytokine networks in colorectal cancer. Nat. Rev. Immunol. *15*, 615–629.

Zelante, T., Iannitti, R.G., Cunha, C., De Luca, A., Giovannini, G., Pieraccini, G., Zecchi, R., D'Angelo, C., Massi-Benedetti, C., Fallarino, F., et al. (2013). Tryptophan catabolites from microbiota engage aryl hydrocarbon receptor and balance mucosal reactivity via interleukin-22. Immunity *39*, 372–385.

Zitvogel, L., Pietrocola, F., and Kroemer, G. (2017). Nutrition, inflammation and cancer. Nat. Immunol. *18*, 843–850.

STAR★METHODS

KEY RESOURCES TABLE

REAGENT or RESOURCE	SOURCE	IDENTIFIER
**Antibodies**		
anti-mouse CD16/CD32 purified	eBioscience	16-0161-86; RRID: AB_468900
APC anti-mouse/human CD11b, clone M1/70	Biolegend	101212; RRID: AB_312795
APC anti-mouse CD3ε, clone 145-2C11	Biolegend	100312; RRID:AB_312677
APC anti-mouse Ly-6G/Ly-6C (Gr-1), clone RB6-8C5	Biolegend	108412; RRID:AB_313377
APC anti-mouse CD19, clone 6D5	Biolegend	115512; RRID:AB_313647
APC anti-mouse TCR β chain, clone H57-597	Biolegend	109212; RRID:AB_313435
PE anti-mouse IL-17A, clone TC11-18H10.1	Biolegend	506904; RRID:AB_315464
APC anti-mouse TCRγδ, clone eBioGL3	eBioscience	17-5711-82; RRID: AB_842757
APC anti-mouse IL-22, clone IL22JOP	eBioscience	17-7222-82; RRID: AB_10597583
PE-Cy7 Rat Anti-Mouse CD90.2, clone 53-2.1	BD Bioscience	561642; RRID:AB_10895975
Alexa Fluor 700 Rat Anti-Mouse CD45, clone 30-F11	BD Bioscience	560510; RRID:AB_1645208
PerCP-Cy5.5 Rat Anti-Mouse CD4, clone RM4-5	BD Bioscience	550954; RRID:AB_393977
BV650 Mouse Anti-Mouse RORγt, clone Q31-378	BD Bioscience	564722
Purified Mouse Anti-E-Cadherin, clone 36/E-Cadherin	BD Bioscience	610181; RRID:AB_397580
Rabbit anti- C.rodentium antiserum	Gift from Dr Gad Frankel	N/A
Goat anti-Rabbit IgG (H+L) Secondary Antibody, Alexa Fluor 555 conjugate	ThermoFicher	A-21429; RRID: AB_2535850
Goat anti-Mouse IgG (H+L) Secondary Antibody, Alexa Fluor 488 conjugate	ThermoFicher	A-11001; RRID: AB_2534069
4′,6-Diamidino-2-phenylindole dihydrochloride (DAPI)	Sigma-Aldrich	D9542
Aryl hydrocarbon receptor polyclonal antibody	Enzo	BML-SA210; RRID:AB_10540536
**Bacterial and Virus Strains**		
C. rodentium infection strain ICC169	Dr.Gad Frankel, Imperial College London	N/A
**Chemicals, Peptides, and Recombinant Proteins**		
Nalidixic acid sodium salt	Sigma-Aldrich	N4382
DPBS, no calcium, no magnesium	ThermoFicher	14190250
Iscove's Modified Dulbecco's Medium	Sigma-Aldrich	I3390
Fetal Bovine Serum	Sigma-Aldrich	F7524
UltraPure 0.5M EDTA	ThermoFisher	15575020
HEPES solution	Sigma-Aldrich	H0887
penicillin/streptomycin	Sigma-Aldrich	P7539
Dithiothreitol (DTT)	ThermoFisher	R0862
DNase I	Sigma-Aldrich	10104159001
Liberase TL	Sigma-Aldrich	5401020001
Percoll	GE HEALTHCARE	17-0891-01
phorbol-12-myristate-13 acetate (PMA)	Sigma-Aldrich	P8139
ionomycin	Sigma-Aldrich	I0634
Brefeldin A	Sigma-Aldrich	B7651
Formaldehyde solution	Sigma-Aldrich	F8775
6-Formylindolo(3,2-b)carbazole (FICZ)	Sigma-Aldrich	SML1489
protease inhibitor cocktail	Sigma-Aldrich	P8340
Dynabeads Protein G	ThermoFisher	10003D

(*Continued on next page*)

Continued		
REAGENT or RESOURCE	SOURCE	IDENTIFIER
Azoxymethane (AOM)	Sigma-Aldrich	A5486
DEXTRAN SULFATE SODIUM SALT	MP Biomedicals	216011080
Advanced DMEM/F-12	ThermoFisher	12634028
L-Glutamine (200 mM)	ThermoFisher	25030149
B-27 Supplement (50X), serum free	ThermoFisher	17504044
N-Acetyl-L-cysteine	Sigma-Aldrich	A9165
murine recombinant EGF	ThermoFisher	PMG8041
SB202190	Sigma-Aldrich	S7067
ALK5 inhibitor A83-01	Bio-Techno	2939
Nicotinamide	Sigma-Aldrich	N0636
TrypLE Express Enzyme (1X), no phenol red	ThermoFisher	12604021
Cultrex PathClear Reduced Growth Factor BME	R&D system	3533-010-02
Critical Commercial Assays		
IL-6 ELISA	Thermofisher	88-7064-88
IL-22 ELISA	Thermofisher	88-7422-88
LIVE/DEAD Fixable Near-IR Dead Cell	Thermofisher	L10119
Fix/Perm buffer	eBioscience	00-5523-00
Click-iT EdU Alexa Fluor 647	Thermofisher	C10424
QIAGEN RNeasy mini kit	QIAGEN	74134
High-Capacity cDNA Reverse Transcription	Thermofisher	4368813
PCR Master Mix	Thermofisher	4318157
ChIP DNA Clean & Concentrator	Zymo Research	D5205
Experimental Models: Cell lines		
HEK293 T cells	ATCC	CRL-1573
L-cells	ATCC	CRL-2648
Experimental Models: Organisms/Strains		
B6.129-*Ahr*^{tm1Bra}/J (Ahr$^{-/-}$)	Jackson Laboratory	Stock No. **002831**
R26^{Cyp1a1}	Schiering et al., 2017	N/A
Villin^{Cre}*R26*$^{LSL\text{-}Cyp1a1}$	Schiering et al., 2017	N/A
Villin^{Cre}*Ahr*$^{fl/fl}$	This paper	N/A
Ahr$^{tm3.1Bra}$/J (*Ahr*$^{fl/fl}$)	Jackson Laboratory	Stock No. 006203
B6.129P2-*Lgr5*$^{tm1(cre/ERT2)Cle}$/J (*Lgr5*$^{Egfp\text{-}Ires\text{-}creErt2}$)	Jackson Laboratory	Stock No. 008875
Oligonucleotides		
B2m	Applied Biosystem	Mm00437762_m1
Il22	Applied Biosystem	Mm01226722_g1
Reg3g	Applied Biosystem	Mm00441127_m1
S100a9	Applied Biosystem	Mm00656925_m1
Muc2	Applied Biosystem	Mm01276696_m1
Car4	Applied Biosystem	Mm00483021_m1
Znrf3	Applied Biosystem	Mm01191453_m1
Rnf43	Applied Biosystem	Mm00552558_m1
Rnf43 promoter: forward 5′ -GTCGCTCACTAGGGGGAGTA-3′; reverse 5′ -TGGTGCACGCACTCACTAAT-3′	Sigma	N/A0
Software and Algorithms		
GraphPad Prism 7.0c	GraphPad Software	https://www.graphpad.com/scientific-software/prism/
FlowJo LLC version 10.4.2	Becton Dickinson	https://www.flowjo.com/solutions/flowjo/downloads

(*Continued on next page*)

Continued		
REAGENT or RESOURCE	SOURCE	IDENTIFIER
Other		
purified diet AIN-93M	TestDiet Europe; IPS Product Supplies	AIN-93M
AIN-93M supplemented with 200mg indole-3-carbinol	TestDiet Europe; IPS Product Supplies	N/A
Indole-3-carbinol	Sigma	I7256

CONTACT FOR REAGENT AND RESOURCE SHARING

Further information and requests for resources and reagents should be directed to and will be fulfilled by the Lead Contact (brigitta.stockinger@crick.ac.uk). There will be restrictions on sharing R26^{Cyp1a1} and *R26$^{LSL\text{-}Cyp1a1}$* mice due to high internal demand.

EXPERIMENTAL MODEL AND SUBJECT DETAILS

Wild-type and *Ahr$^{-/-}$* mice as well as R26^{Cyp1a1}, *Villin^{Cre}Ahr$^{fl/fl}$*, *Villin^{Cre}R26$^{LSL\text{-}Cyp1a1}$* mice and *Lgr5$^{Egfp\text{-}Ires\text{-}creErt2}$* (Barker et al., 2007) (all on a C57BL/6 background) were bred and maintained in individually ventilated cages at the Francis Crick Institute, under specific pathogen-free conditions according to the protocols approved by the UK Home Office and the ethics committee (AWERP) of the Francis Crick Institute. Littermates of the same sex were randomly assigned to experimental groups in an age range of 5-9 weeks unless otherwise specified.

METHOD DETAILS

Generation of BM chimeras

BM cells were obtained from femurs and tibias of donor mice. Sub-lethally irradiated WT and *Ahr$^{-/-}$* recipient mice were reconstituted by intravenous injection of 2.5x10^6 BM cells.

Infection with Citrobacter rodentium

For C. rodentium infection a single colony of strain ICC169 was transferred to Luria–Bertani (LB) broth supplemented with nalidixic acid (Nal) to a final concentration of 50 μg/ml for selection and grown to log phase followed by centrifugation and resuspension in PBS. Mice were orally gavaged with 200 μL of PBS containing 2x10^8 C. rodentium. To determine bacterial load, intestinal tissue pieces or faecal pellets were weighed and homogenized in sterile PBS and serial dilutions were plated onto LB agar plates with selective marker Nal for measurement of colony-forming units (CFU).

Isolation of lamina propria cells and flow cytometry

Colon were cut open longitudinally and incubated in wash buffer (IMDM 1%FCS, 5mM EDTA, 10mM HEPES, penicillin/streptomycin, and 2mM DTT) for 20min at 37°C with 200rpm shaking. Colon tissue was collected, cut into small pieces and incubated in digestion buffer (IMDM supplemented with 1% FCS, 10mM HEPES, penicillin/streptomycin, 50 μg/ml DNase I (Roche)) containing 0.4mg/ml Liberase TL (Roche) and incubated for 30min at 37°C with 200rpm shaking. Single cell suspensions from colon were further subjected to 40% Percoll (Amersham) density gradient centrifugation to remove debris. For surface staining, cell suspensions the lamina propria were incubated with anti-CD16/CD32 (eBioscience) and fixable live/dead cell dye (ThermoFisher) followed by staining with antibodies against CD11b, CD3, TCRγδ, Gr1, CD11b, CD19, TCR-β (all BioLegend) and Thy1.2, CD45, CD4 (all BD Biosciences). For intracellular staining, single cell suspensions were re-stimulated for 2hrs and 15min in the presence of 1ng/ml phorbol-12-myristate-13 acetate (PMA), 1 μg/ml ionomycin and 10 μg/ml Brefeldin A (all Sigma), washed and stained for surface markers as described above. Cell were then fixed in eBioscience Fix/Perm buffer or 4% formaldehyde (for preservation of eYFP fluorescence) for 30min on ice followed by permeabilization in eBioscience permeabilization buffer for 45min in the presence of antibodies against IL-17A, IL-22, (all ebioscience) and RORγt (BD Biosciences). Cells were acquired with a BD Fortessa and analysis was performed with FlowJo v10 (Tree Star) software.

Colon explant cultures

Intestinal tissue pieces (0.5-1cm length) were cultured for 24 hours in complete IMDM medium. IL-22 cytokine levels in the supernatants were determined by ELISA (eBioscience) and concentrations were normalized to the weight of the explants.

Immunohistochemistry and Immunofluorescence Staining

Tissues were fixed in 4% phosphate-buffered formaldehyde solution (Fisher Scientific) for 24 hours. Fixed tissue sections were deparaffinised and antigen retrieval performed in 0.01M sodium citrate buffer. Slides were blocked with goat serum, stained with mouse

anti-E-cadherin (BD, 610181) and rabbit anti- C.rodentium antiserum followed by staining with secondary antibodies (AF555- conjugated goat-anti-rabbit and AF488-conjugated goat-anti-mouse from ThermoFisher). Slides were further stained with DAPI (Sigma) and mounted in Fluoromount-G (SouthernBiotech) and visualized using a Leica Confocal SP5- Invert microscope. Image analysis was performed in ImageJ.

Quantitative Real-time Polymerase Chain Reaction

RNA was isolated from organoids cultures using the QIAGEN RNeasy mini kit, according to the manufacturer's instructions. One microgram total RNA was reverse-transcribed using the ThermoScript RT-PCR System kit (Applied biosystem). The cDNA served as a template for the amplification of genes of interest and the housekeeping genes (*B2m*) by real-time quantitative PCR, using TaqMan Gene Expression Assays (Applied Biosystems), universal PCR Master Mix (Applied Biosystems) and the ABI-PRISM 7900 sequence detection system (Applied Biosystems). mRNA expression levels were determined using the ΔCt method, relatively to the level of *B2m* (beta-2-microglobulin) gene expression.

The following probes (Applied Biosystems) have been used: *B2m* (Mm00437762_m1), *Il22* (Mm01226722_g1), *Reg3g* (Mm00441127_m1), *S100a9* (Mm00656925_m1), *Muc2* (Mm01276696_m1), *Car4* (Mm00483021_m1), *Znrf3* (Mm01191453_m1), *Rnf43* (Mm00552558_m1).

Chromatin immunoprecipitation

Single cell suspensions from colon were incubated with 5nM of FICZ for 3h at 37°C. 3.10^6 cells were crosslinked with 1% paraformaldehyde and lysed in 1 mL of lysis buffer (1% SDS, 10 mM EDTA, 50 mM Tris–HCl, pH 8) containing protease inhibitor cocktail (Roche). Chromatin was then sheared by sonication, and centrifuged at 14,000 rpm for 10 min at 4°C; 5% of sonicated cell extract was kept as input. Supernatants were then diluted in dilution buffer (1% Triton X-100, 150 mM NaCl, 2 mM EDTA, 20 mM Tris–HCl, pH 8) and immunoprecipitated overnight at 4°C with 2 μg of anti-AhR antibody (BML-SA210, Enzo) or 5 mg of anti-ARNT antibody (sc-8076, C-19, Santa Cruz Biotechnology). Protein G Dynabeads (Life Technologies) were then added to the cell extract for 4h at 4°C. Samples were washed once in low-salt buffer (0.1% SDS, 1% Triton X-100, 2 mM EDTA, 150 mM NaCl, 20 mM Tris–HCl, pH 8), once in high-salt buffer (same as low salt except for 500 mM NaCl), once in LiCl wash buffer (0.25 M LiCl, 1% NP40, 1% sodium deoxycholate, 1 mM EDTA, 10 mM Tris–HCl, pH 8) and twice with 10 mM Tris–HCl 1mM EDTA. Protein/DNA complexes were eluted in elution buffer (1% SDS, 100 mM NaHCO3) at room temperature for 30 min followed by 2 min at 65°C. Crosslinking was reversed by the addition of NaCl (200 mM final) and incubation overnight at 65°C. After RNase and proteinase K treatment, DNA fragments were purified using the Chip DNA Clean and concentrator kit (Zymo Research) and analyzed by quantitative PCR and by normalization relative to input DNA amount. The following primers were used for the Rnf43 promoter: forward 5′ -GTCGCTCACTAGGGG GAGTA-3′; reverse 5′ -TGGTGCACGCACTCACTAAT-3′.

Azoxymethane–DSS model of colorectal tumorigenesis

For spontaneous tumor progression model, seven weeks old male mice were injected intraperitoneally with 10cmg of azoxymethane (AOM, Sigma) per kg body weight once a week for 6 weeks. After 120 days, the mice were sacrificed to evaluate tumor development. For chronic inflammation driver tumor progression, the mice were injected with 10mg of AOM. After 5 days, 1% DSS (MP Biomedicals) was given in the drinking water for 6 days followed by regular drinking water for 2 weeks. This cycle was repeated once more and mice were killed on day 60.

Organoid Culture

Mouse organoids were established and maintained at 37C as three-dimensional spheroid culture in Matrigel (R&D system) from isolated crypts collected from the colon of C57BL/6, $Ahr^{-/-}$ and $R26^{Cyp1a1}$ mice. The basic culture medium (ENR) contained advanced DMEM/F12 supplemented with penicillin/streptomycin, 10mM HEPES, 2mM Glutamax, B27 (all from Life Technologies) and 1mM N-acetylcysteine (Sigma) supplemented with murine recombinant EGF (life technologies), R-spondin1-CM (Trevigen) (20% final volume) and Noggin-CM (20% v/v). For mouse large intestine, crypts were cultured in 'WENR' medium containing 50% WNT3a-conditioned medium supplemented with SB202190 (10cμM, Sigma), ALK5 inhibitor A83-01 (500cnM, Tocris Bioscience) and nicotinamide (10 mM, Sigma) or is some cases in WEN medium lacking R-spondin 1. The percentage of viable organoids was determined using OrganoSeg software (Borten et al., 2018).

EdU Click-iT cell cycle flow cytometry assay

Cells were incubated with 10 μM EdU for 24 hours. Epithelial spheroids were isolated from 3D Matrigel culture in PBS-EDTA and trypanized for 5 minutes at 37°C with TrypLE. The EdU Click-iT Flow Cytometry Assay kit (Invitrogen) was then used to determine the percentage of S-phase cells according to the manufacturer's instructions. Flow cytometry data was analyzed using FloJo V10 software.

Dietary intervention

For diet studies mice were fed purified diet AIN-93M (TestDiet-IPS) or AIN-93M supplemented with 200ppm indole-3-carbinol (Sigma). Mice were put on purified diets for at least 2 weeks and maintained on the purified diets throughout the duration experiments.

QUANTIFICATION AND STATISTICAL ANALYSIS

For comparisons between two groups unpaired, two-tailed Student's t test was used or when appropriate a two-way ANOVA with Dunnett's post-test. For the comparison of three groups a one-way ANOVA followed by Tukey multiple comparison test was performed. All statistical analysis was calculated in Prism (GraphPad 7). 'n' represents number of biological replicates and is defined in the figure legends.

Immunity, Volume *49*

Supplemental Information

The Environmental Sensor AHR Protects from Inflammatory Damage by Maintaining Intestinal Stem Cell Homeostasis and Barrier Integrity

Amina Metidji, Sara Omenetti, Stefania Crotta, Ying Li, Emma Nye, Ellie Ross, Vivian Li, Muralidhara R. Maradana, Chris Schiering, and Brigitta Stockinger

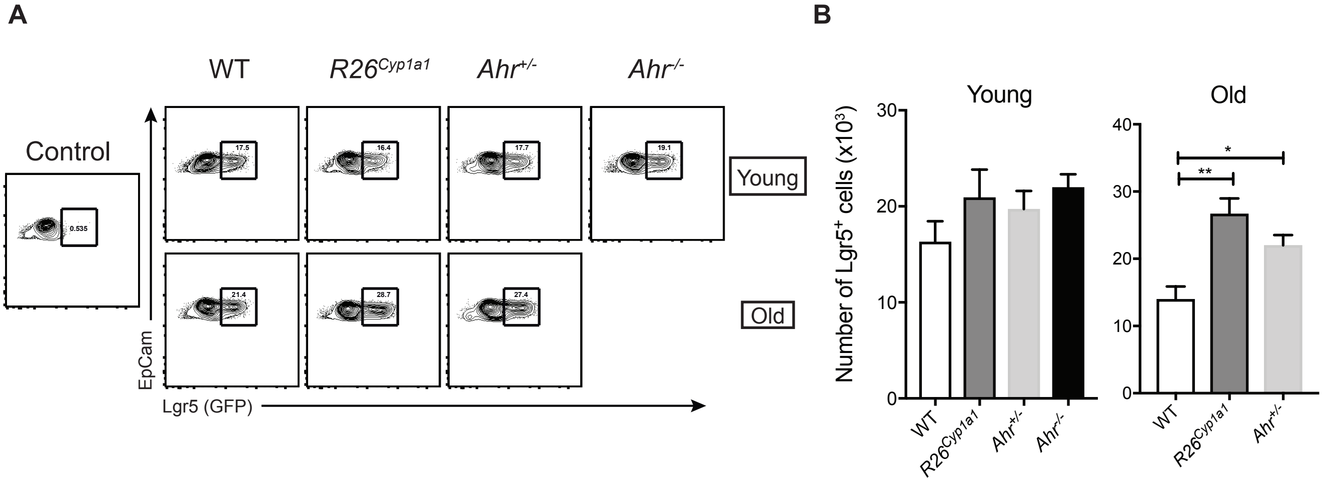

Figure S1. Expansion of Lgr5⁺ cells with age, related to Figure 3.
(A) Flow cytometry analysis of Lgr5 expression in EpCam⁺ cells from the colon of the indicated mice at steady state.
(B) Percentage of Lgr5⁺ cells at steady state in young (5-8 weeks of age) and old (14-16 weeks of age) mice.
Error bars, mean + s.e.m. * $P < 0.05$, ** $P < 0.01$, *** $P < 0.001$, as calculated by one-way ANOVA with Tukey post-test.

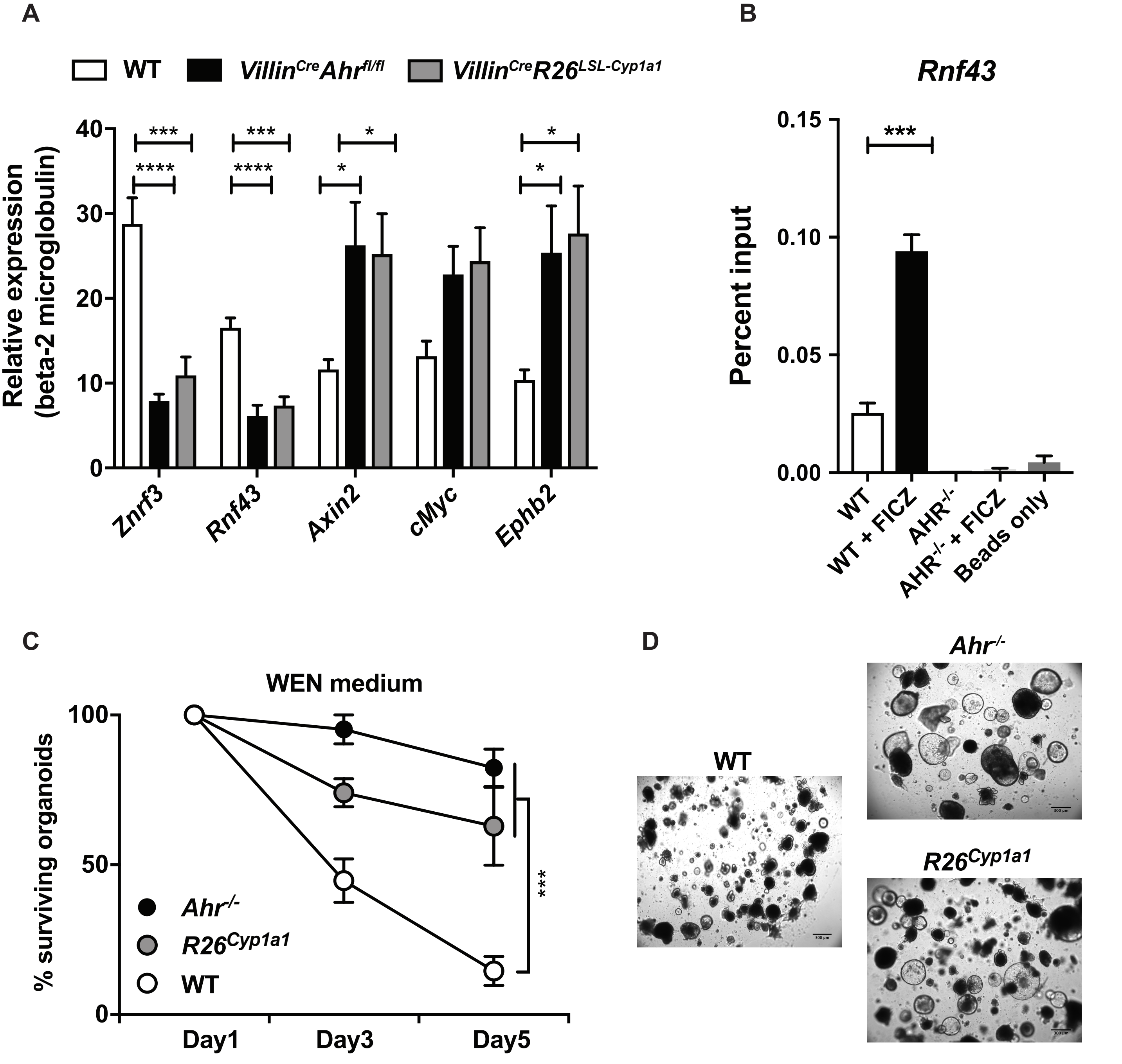

Figure S2. Dysregulated feedback signaling in the Wnt-β-Catenin pathway of AHR mutant mice, related to Figure 4.
(A) qPCR analysis of WNT negative regulators (*Znrf3* and *Rnf43*) and WNT target genes (*Axin2*, *cMyc*, and *Ephb2*) in the colon of 14 weeks old mice (WT, n=5; *Villin^{Cre}Ahr$^{fl/fl}$*, n=5; *Villin^{Cre}R26$^{LSL-Cyp1a1}$*, n=5) at steady state. (B) Chromatin immunoprecipitation (ChIP) analysis of AHR interaction with *Rnf43* promoter in WT, WT treated with FICZ, *Ahr$^{-/-}$*, and *Ahr$^{-/-}$* treated with FICZ in stem cells organoids. (C) Percentage surviving organoids following culture in WEN medium (without R-spondin1) over 5 days calculated using OrganoSeg software. (D) Representative images of WT, *Ahr$^{-/-}$* and *R26^{Cyp1a1}* organoids after 5 days in WEN medium. Error bars, mean + s.e.m. *$P < 0.05$, **$P < 0.01$, ***$P < 0.001$, as calculated by one-way ANOVA with Tukey post-test.

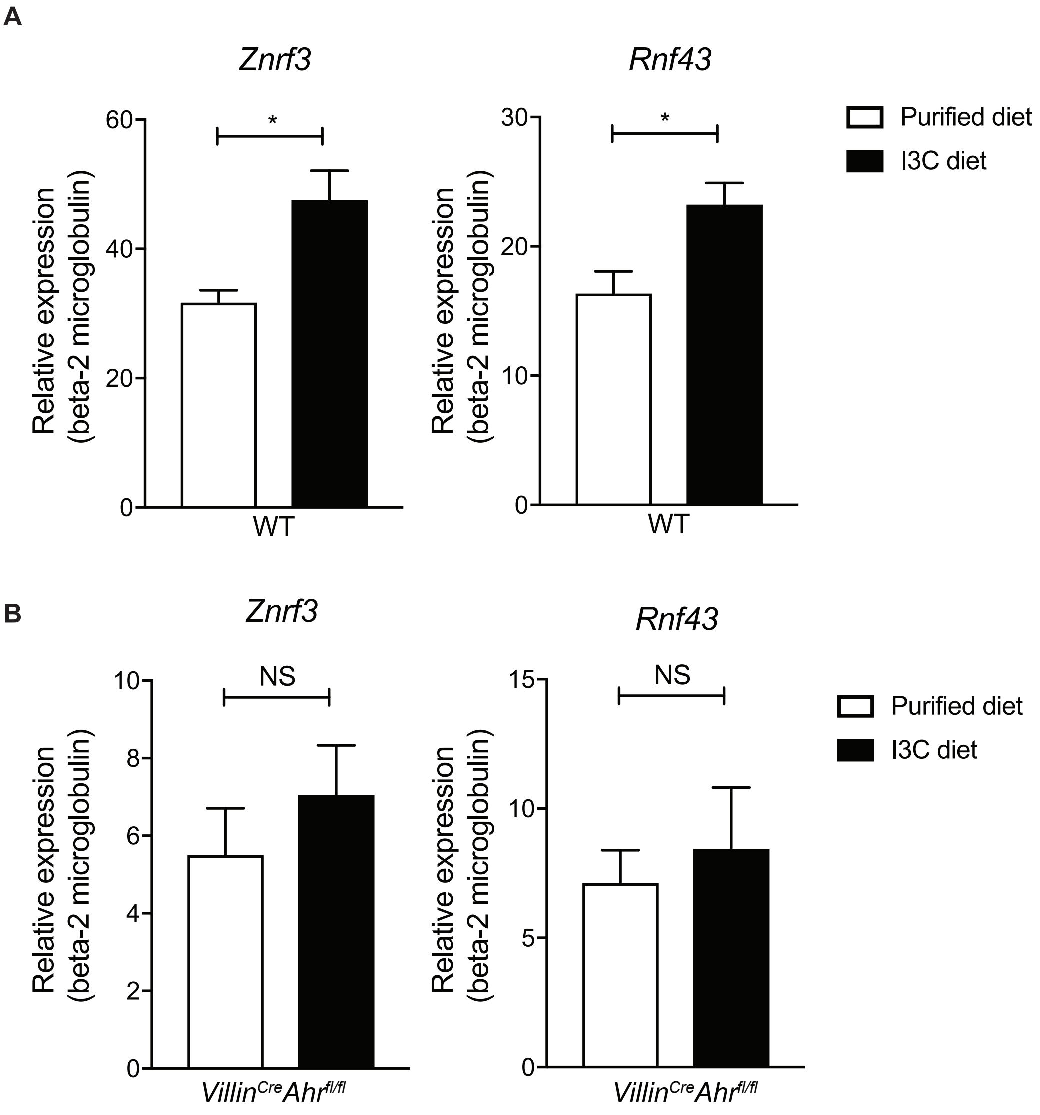

Figure S3. I3C diet increases WNT pathway regulator expression in WT but not $Villin^{Cre}Ahr^{fl/fl}$ mice, related to Figure 5. (A) qPCR analysis of WNT negative regulators (*Znrf3* and *Rnf43*) in the colon of WT, n=5 after AOM/DSS fed purified or I3C diet. (B) qPCR analysis of WNT negative regulators (*Znrf3* and *Rnf43*) in the colon of $Villin^{Cre}Ahr^{fl/fl}$, n=5 after AOM/DSS fed control or I3C diet. Error bars, mean + s.e.m. * $P < 0.05$, ** $P < 0.01$, *** $P < 0.001$, as calculated by unpaired t-test.
